# Awareness of Rhythm Patterns in Speech and Music in Children with Specific Language Impairments

**DOI:** 10.3389/fnhum.2015.00672

**Published:** 2015-12-22

**Authors:** Ruth Cumming, Angela Wilson, Victoria Leong, Lincoln J. Colling, Usha Goswami

**Affiliations:** Department of Psychology, Centre for Neuroscience in Education, University of CambridgeCambridge, UK

**Keywords:** SLI, phonology, auditory processing, rise time

## Abstract

Children with specific language impairments (SLIs) show impaired perception and production of language, and also show impairments in perceiving auditory cues to rhythm [amplitude rise time (ART) and sound duration] and in tapping to a rhythmic beat. Here we explore potential links between language development and rhythm perception in 45 children with SLI and 50 age-matched controls. We administered three rhythmic tasks, a musical beat detection task, a tapping-to-music task, and a novel music/speech task, which varied rhythm and pitch cues independently or together in both speech and music. Via low-pass filtering, the music sounded as though it was played from a low-quality radio and the speech sounded as though it was muffled (heard “behind the door”). We report data for all of the SLI children (*N* = 45, IQ varying), as well as for two independent subgroupings with intact IQ. One subgroup, “Pure SLI,” had intact phonology and reading (*N* = 16), the other, “SLI PPR” (*N* = 15), had impaired phonology and reading. When IQ varied (all SLI children), we found significant group differences in all the rhythmic tasks. For the Pure SLI group, there were rhythmic impairments in the tapping task only. For children with SLI and poor phonology (SLI PPR), group differences were found in all of the filtered speech/music AXB tasks. We conclude that difficulties with rhythmic cues in both speech and music are present in children with SLIs, but that some rhythmic measures are more sensitive than others. The data are interpreted within a “prosodic phrasing” hypothesis, and we discuss the potential utility of rhythmic and musical interventions in remediating speech and language difficulties in children.

## Introduction

There is growing interest in a range of disciplines over whether language processing and music processing draw on shared neural resources (e.g., Patel, 2010; Rebuschat et al., 2012, for overviews). In developmental psychology studies, this interest is fuelled in part by the belief that music may offer novel interventions to help children with language learning impairments (e.g., Koelsch et al., 1999; Besson et al., 2007; Elmer et al., 2012). Here we explore the awareness of rhythm patterns in speech and music in children with speech and language impairments (SLIs), adopting a theoretical focus drawn from the relationship between children's sensitivity to amplitude rise time (ART) and the accuracy of their neural entrainment to amplitude modulations (AMs) in the speech signal [Temporal Sampling (TS) theory, see Goswami, 2011, 2015, for overviews]. Based on temporal sampling theory, we have proposed a new perceptual hypothesis to explain the etiology of SLIs across languages, the “prosodic phrasing hypothesis” (Cumming et al., 2015). The prosodic phrasing hypothesis proposes that an auditory difficulty in the accurate processing of ARTs (Fraser et al., 2010; Beattie and Manis, 2012) and sound duration (Corriveau et al., 2007; Richards and Goswami, 2015) causes children with SLIs to have difficulties in detecting the prosodic or rhythmic phrasing of given utterances. Theoretically, prosodic patterning is considered to be the skeletal beat-based structure upon which human language processing builds (for both phonology and syntax, see e.g., Gleitman et al., 1988; Frazier et al., 2006; Goswami and Leong, 2013).

Although prosodic phrasing in adult-directed speech is not periodic and therefore difficult to conceive of as beat-based, it is important to note that *child-directed speech* is often overtly rhythmic. An example is the English “nursery rhyme,” which typically has strong trochaic or iambic beat structure (Gueron, 1974). ART (or “attack time” for musical notes) is perceptually critical for perceiving beat structure (patterns of beats, see Goswami et al., 2013). The time to peak amplitude modulation (i.e., ART) governs “P-centre” perception, the subjective moment of occurrence (or “perceptual centre”) of a beat, whether in language (a syllable beat), or in music (Scott, 1993). Leong and Goswami (2015) modeled the AM structure of English nursery rhymes using an AM phase hierarchy approach drawn from neural models of oscillatory speech encoding (see also Leong and Goswami, 2014). The acoustic modeling revealed three nested temporal tiers of AMs in English nursery rhymes, at approximately 2, 5, and 20 Hz. These three modulation rates enabled the model to extract phonological structure (prosody, syllables, and the onset-rime syllable division). Nursery rhymes are inherently rhythmic, but when adults deliberately spoke them rhythmically (in time with the regular beat of a pacing metronome), then the model's success in extracting phonological units improved significantly. For freely-produced nursery rhymes, the Spectral-Amplitude Modulation Phase Hierarchy (S-AMPH) model detected 72% of strong-weak syllable alternations successfully, compared to 95% when the nursery rhymes were spoken to a metronome beat. Regarding syllable finding, for freely-produced nursery rhymes the model detected 82% of syllables successfully, compared to 98% success for deliberately timed nursery rhymes. The corresponding figures for onset-rime discrimination were 78 vs. 91% (see Leong and Goswami, 2015). The S-AMPH acoustic modeling supports a series of behavioral studies across languages showing significant relationships between ART discrimination and phonological development in children (Goswami, 2011, 2015 for summaries).

The prosodic phrasing hypothesis for SLI (Cumming et al., 2015) builds on these documented relationships between ART, AMs and phonological development. It suggests that grammatical impairments in children with SLIs arise in part from their perceptual difficulties in discriminating ART and duration. These perceptual impairments make it more difficult for SLI children to benefit from rhythmic patterning in language and hence to extract the phonological structure that often supports syntax (see Cumming et al., 2015). Accordingly, in the current study we investigate the sensitivity of children with SLIs to rhythmic patterning in language and music. Accurate behavioral assessments should provide a guide regarding whether musical rhythmic remediation might support the perception of speech rhythm in children with SLIs, thereby facilitating the perception of prosodic phrasing.

Children with SLIs have been shown to be impaired in tapping to a metronome beat, at temporal rates that approximately reflect the occurrence of stressed syllables in speech (focused around 2 Hz, see Corriveau and Goswami, 2009). The temporal sampling framework proposes that the stressed syllable rate is a foundational AM rate for language learning. Across languages, adult speakers produce stressed syllables roughly every 500 ms (Dauer, 1983), and stressed syllables are cued by large changes in ART. These regular 2 Hz ARTs appear to provide crucial perceptual cues to prosodic structure, providing a beat-based skeleton for language learning (see Goswami and Leong, 2013; Goswami, 2015, for detail). Infants use prosody to segment the speech stream across languages (Jusczyk et al., 1999). Perceptual difficulties in discriminating AM rates could be associated with atypical oscillatory neural entrainment to the speech signal for affected infants, with associated difficulties in perceiving prosodic structure and consequently in extracting phonological and morphological information from speech. The literature on behavioral motor synchronization to the beat is a large one (Repp, 2005; Repp and Yi-Huang, 2013), but motor variability in synchronization to the beat tasks at rates around 2 Hz are related to language impairments and to phonological/literacy impairments in prior studies (Thomson and Goswami, 2008; Corriveau and Goswami, 2009). Accordingly, Goswami has argued that neural entrainment and phase-phase alignment of neuronal oscillations across motor and sensory areas may offer a developmental framework for considering shared neural resources in processing language and music (e.g., Goswami, 2012a,b).

Specifically, the temporal sampling model would predict that training children with SLIs to match rhythm in music, which is overt and is typically supported by multiple cues in the melody, with rhythm in speech, may benefit linguistic processing for children with SLIs.

Studies showing links between music processing and language processing are indeed beginning to emerge in the developmental literature. For example, Francois et al. (2012) gave typically-developing (TD) French-speaking children either a music intervention or a painting intervention when they were aged 8 years, and tested their speech segmentation performance. The speech segmentation task utilized an unbroken sung sequence of triples of nonsense syllables produced without prosodic phrasing, which lasted for 5 min (“gimysy-sipygy-pogysi-pymiso… ”; hyphens segment the individual nonsense “words,” although no actual physical pauses occurred). Children then made a forced-choice response concerning whether a spoken item had been part of the artificial language or not [e.g., gimysy (yes) vs. sysipy (no)]. Francois et al. reported that while both groups of children were at chance in this task in a pretest, the music intervention children were significantly better at this task than the painting intervention children after both 1 and 2 years of intervention. A second study, also with TD children, asked English-speaking participants aged 6 years to make same-different judgements about tone sequences arranged in different metrical patterns, and to judge whether short monotonic melodies had the same or different rhythms (Gordon et al., 2015). The children were also given an expressive grammar test requiring them to describe pictures using different morpho-syntactic constructions. Gordon and colleagues reported a significant correlation between the children's rhythm discrimination scores and their accuracy in the expressive grammar test, which remained significant when individual differences in phonological awareness were controlled. Gordon et al. concluded that there was a robust relationship between rhythmic and syntactic abilities in TD children.

Regarding atypical development, Przybylski et al. (2013) investigated the utility of acoustic synchronization to the beat for supporting grammatical performance by French children with SLIs. They gave 9-year-olds a grammaticality judgment task, and manipulated whether a musical prime supported the grammaticality judgements. The musical prime either had a regular beat pattern based on a tempo of 500 ms (2 Hz) or an irregular beat pattern with varying tempo, produced by tam tams and maracas. Children judged sentences like “Le camera filme les danseurs” (gender agreement violation, as it should be “La camera”). Przybylski and colleagues reported that hearing the regular musical prime significantly improved grammaticality judgements, for both the children with SLI and for TD control children, and interpreted these improvements using temporal sampling theory. They argued that the predictable metrical structure of the music might entrain internal oscillators that supported temporal segmentation of speech input at the sentence level (see Large and Jones, 1999). In temporal sampling theory, these internal oscillators would be auditory cortical networks entraining to the amplitude modulation hierarchy in speech (see Goswami, 2011, 2015).

Regarding phonological awareness, Dellatolas et al. (2009) asked 1028 TD French-speaking children aged 5–6 years to produce 21 rhythmic patterns modeled by the experimenter by tapping with a pencil on a table. Dellatolas et al. reported that individual differences in rhythmic performance were a significant predictor of reading at age 7–8 years (2nd grade), even after controlling for attention and linguistic skills. Indeed, the rhythm reproduction task showed a normal distribution, and a linear relationship with attainment in reading. In a phonological training study with German-speaking children, Degé and Schwarzer (2011) offered preschoolers (4- to 5-year-olds) either musical training, a sports intervention, or phonological training. The musical training included joint singing, joint drumming, rhythmic exercises, metrical training, dancing and rudimentary notation skills, and was given to small groups for 10 min daily for 20 weeks. Degé and Schwarzer reported significant improvements in phonological awareness for the music group, equivalent to the improvements made by the phonological awareness training group. Both groups showed significantly more improvement than the children receiving the sports intervention.

Most recently, a study by Bhide et al. (2013) explored the possible benefits of a music and rhythm intervention based on temporal sampling theory for phonological and reading development. The intervention related musical beat patterns to the theoretically-related linguistic factors of syllable stress, syllable parsing and onset-rime awareness. Children aged 6–7 years participated in activities like bongo drumming to varied beat structures in music, making rhythm judgements with different tempi, marching and clapping to songs, learning poetry and playing chanting/hand-clap games. The children showed significant improvements in phonological awareness, reading and spelling, with large effect sizes (e.g., *d* = 1.01 for rhyme awareness). Circular statistics showed that individual improvements in motor synchronization to the beat and reading were significantly related, supporting temporal sampling theory.

In the current study, we extended temporal sampling theory to SLI children. We gave 45 children with SLIs a set of non-speech rhythm tasks, in order to explore their sensitivity to beat patterns in music. The SLI children were known to have significant impairments in processing syllable stress related to auditory impairments in perceiving ARTs and sound duration (see Cumming et al., 2015). The first non-speech rhythm task measured sensitivity to patterns of musical beat distribution, and was originally devised for children with developmental dyslexia (Huss et al., 2011; Goswami et al., 2013). Children with dyslexia aged 10 years showed significant difficulties in the task, scoring on average 63% correct compared to 84% correct for their TD controls. Performance in the musical beat processing task along with age and IQ explained over 60% of the variance in single word reading in the dyslexic sample. The second non-speech task was a tapping-to-music task. The music was varied to have either 3/4 time or 4/4 time, with the former expected to be more difficult. The aim was to see whether motor synchronization to the beat would be easier for children with SLIs when a rich musical stimulus was used (a metronome beat was used by Corriveau and Goswami, 2009). We also created a novel task measuring children's sensitivity to pitch contour and rhythm patterns in both speech and music. This was an AXB task in which matching to the standard (X) was required on the basis of either rhythm, pitch contour or both rhythm and pitch contour.

Overall, we expected to find difficulties in perceiving musical rhythm in our children with SLIs, as the children had known impairments in perceiving ART and duration (Cumming et al., 2015). Studies with dyslexic children have previously shown that ART sensitivity is related to musical beat perception, in both cross-sectional and longitudinal assessments (Huss et al., 2011; Goswami et al., 2013). Of interest here was whether difficulties in rhythmic processing in the same task would be less pronounced or greater for children with SLIs compared to children with dyslexia, and whether the processing of pitch contours as well as rhythm would be affected in music and/or speech for children with SLIs. As melody depends on both rhythm and pitch, greater facility with the music tasks compared to the speech tasks would support the use of language interventions based on music for children with SLIs.

## Methods

### Participants

Ninety-five children aged on average 9 years 6 months participated in this study, of whom 45 were referred by their schools as having a specific language impairment (SLI). All participants and their guardians gave informed consent, and the study was approved by the Psychology Research Ethics Committee of the University of Cambridge. Only children who had no additional learning difficulties (e.g., dyspraxia, ADHD, autistic spectrum disorder, dyslexia) and English as the first language spoken at home were included. The absence of additional learning difficulties was based on the reports of teachers and speech and language therapists in schools, and our own testing impressions of the children. All children received a short hearing screen using an audiometer. Sounds were presented in both the left and the right ear at a range of frequencies (250, 500, 1000, 2000, 4000, 8000 Hz), and all children were sensitive to sounds within the 20 dB HL range. Forty-five of the children (31 male, 14 female; mean age 9 years, 6 months; range 6 years 4 months to 12 years 1 month) either had a statement of SLI from their local education authority, or had received special help for language via the teacher(s) with responsibility for special educational needs in school, and/or showed severe language deficits according to our own test battery. These children (SLI group) were drawn from a number of schools via language support units in the schools, referral to the study by speech and language therapists or referral by teachers with responsibility for special educational needs (note that SLI is more common in boys, with a ratio of ~4:1). All children with SLI were assessed experimentally using two expressive and two receptive subtests of the Clinical Evaluation of Language Fundamentals-3 (CELF-3; Semel et al., 1995), and were included in the study if they scored at least 1 SD below the mean on two or more of these subtests. Individual standardized scores of the children in the SLI group for the four CELF-3 subtests administered, as well as receptive vocabulary as measured by the British Picture Vocabulary Scales (BPVS, Dunn, Dunn et al., 1982), and nonverbal IQ as measured by the Wechsler Intelligence Scales for Children (WISC-III; Wechsler, 1992) or Raven's Standard Progressive Matrices (Plus version, Raven, 2008), are shown in Table 1. The table also shows single-word reading and spelling scores on the British Ability Scales (BAS; Elliott et al., 1996) and the Test of Word Reading Efficiency (TOWRE; Torgesen et al., 1999).

**Table 1:** **Participant details for the children with SLIs, showing sub-group membership**.

**SLI sub-group**	**BPVS^a^**	**CELF expressive^b^**		**CELF receptive**		**NIVQ^c^**	**Reading and spelling**		
		**FS**	**SA/WS**	**CD**	**SR/SS**		**TOWRE ^d^**	**BAS read^e^**	**BAS spell^e^**
Pure	107	8	5	9	6	131	101	114	118
Pure	78	5	6	12	7	125	111	117	127
Pure	96	11	5	12	6	119	118	106	99
Pure	85	4	4	3	4	110^f^	89	105	87
Pure	93	7	7	7	7	110^f^	101	99	109
Pure	80	5	7	6	4	105^f^	95	86	89
Pure	92	5	6	4	10	105^f^	87	78	91
Pure	98	6	5	11	10	103	90	108	105
Pure	104	11	6	6	10	100	101	112	106
Pure	106	5	5	4	3	97	114	118	118
Pure	84	4	4	5	5	95^f^	103	106	131
Pure	89	3	6	4	5	90^f^	96	103	90
Pure	90	7	5	3	11	90^f^	N/A	86	93
Pure	104	9	4	7	5	88	119	110	105
Pure	100	8	3	6	4	81	93	94	90
Pure	101	3	4	6	5	80^f^	93	86	61
PPR	77	3	4	8	7	115^f^	68	74	66
PPR	101	3	6	6	11	112	69	82	68
PPR	86	3	3	6	4	105^f^	80	79	91
PPR	81	3	4	3	5	100	60	55	55
PPR	72	2	4	4	5	97	64	69	60
PPR	90	6	4	8	13	97	80	82	78
PPR	74	3	5	5	5	95^f^	79	85	77
PPR	78	3	3	3	5	95^f^	82	81	79
PPR	89	2	4	6	12	94	87	81	78
PPR	97	5	3	3	3	94	N/A	55	55
PPR	107	3	7	6	6	94	72	80	78
PPR	76	2	3	6	4	91	63	69	59
PPR	90	3	3	3	4	85	56	64	63
PPR	91	5	3	6	6	81	65	74	63
PPR	90	3	3	6	6	80^f^	71	79	76
	87	1	6	1	2	75	N/A	55	59
	100	8	4	8	6	75^f^	101	96	89
	85	3	3	6	6	75^f^	74	77	79
	89	1	1	1	6	75^f^	54	64	69
	83	8	3	3	4	70^f^	94	92	101
	80	3	3	3	4	75^f^	102	96	97
	76	3	3	4	3	70^f^	54	67	65
	73	3	4	3	4	70^f^	54	56	62
	87	3	2	4	4	65^f^	62	70	74
	86	5	4	10	12	65^f^	68	71	75
	72	3	3	3	3	60^f^	N/A	55	55
	90	3	3	3	3	57	N/A	55	55
	59	3	3	3	3	55^f^	N/A	55	55
	64	3	3	6	5	55^f^	84	82	90
Mean (sd)	87.49 (11.38)	4.44 (2.44)	4.13 (1.39)	5.38 (2.62)	5.84 (2.78)	96.05 (49.56)	83.44 (33.58)	82.48 (22.40)	82.00 (20.65)

*N/A means score not available, either as the child was absent or as the child refused to try the non-word component of the TOWRE, which is a timed test*.

a *British Picture Vocabulary Standard Score (M = 100, SD = 15)*.

b *Clinical Evaluation of Language Fundamentals (CELF) Expressive and Receptive Sub-tests (M = 10, SD = 3)*.

c *Higher Standard Score of WISC or Ravens (M = 100, SD = 15)*.

d *Test of Word Reading Efficiency combined Standard Score (M = 100, SD = 15)*.

e *British Ability Scales Standard Score (M = 100, SD = 15)*.

f *Ravens SS shown instead of WISC SS*.

Note that in our prior studies of ART and beat perception in music in dyslexia (e.g., Goswami et al., 2013), only children with a diagnosis of dyslexia and no history of speech or language impairments were studied. Here, we studied children with a diagnosis of SLI and no history or diagnosis of reading impairments. Nevertheless, as indicated on Table 1, a number of the 45 children with SLI did show impaired reading on our test battery. Table 1 also shows that IQ varied greatly within the SLI group. Therefore, from this sample of 45 SLI children, we created two sub-groups with intact IQ. Following Fraser et al. (2010), children with SLI were regarded as having non-verbal IQ within the normal range if they scored 80 or above on at least one of the two non-verbal measures (WISC, Ravens). One sub-group comprised a sample of children with pure SLI and no IQ or reading difficulties (*N* = 16, 11 boys), hereafter the “Pure SLI” group. The second sub-group (*N* = 15, 4 boys) comprised a separate sample of SLI children with preserved IQ but reading difficulties, defined as having a SS < 85 on at least two of the standardized reading and spelling tests used. These children also showed phonological difficulties on the experimental measures of phonological processing used (described below), hence hereafter they are termed the “SLI PPR” (poor phonology and reading) group. Note that the SLI PPR children would not qualify for a diagnosis of developmental dyslexia because of their spoken language impairments. As there is no theoretical reason to expect auditory processing skills to vary with I.Q. (see Kuppen et al., 2011), we analyse data for the entire sample of SLI children as well as for these two sub-groups (Pure SLI, SLI PPR).

Fifty chronological age (CA) matched control children from the same schools as the SLI children also participated in the study. These comprised children who returned consent forms and who were close to individual SLI participants in age. The control group included 21 males and 29 females, with a mean age of 9 years, 4 months, range 6 years 4 months to 11 years 8 months). By selecting control children with non-verbal IQ and reading in the normal range, we created a matched sample of typically-developing children for the Pure SLI group (*N* = 16) and for the SLI PPR group (*N* = 15). Group matching for the standardized ability tasks is shown in Table 2 for these two SLI sub-groupings. Table 2 also includes performance on experimental tests of phonology (see below), to indicate that there were no phonological impairments in the Pure SLI group.

**Table 2:** **Participant characteristics by matched sub-group**.

	**Pure SLI *N* = 16**	**Controls *N* = 16**	***F*_(1, 31)_**	**SLI PPR *N* = 15**	**Controls N = 15**	***F*_(1, 29)_**
Age in months	109.4 (20.8)	106.6 (17.1)	0.2	115.5 (14.0)	107.6 (17.2)	1.9
CELF REC SS^a^^,^ ^b^	12.9 (4.2)	21.3 (4.0)	32.9^***^	11.7 (4.3)	21.2 (4.2)	37.7^***^
CELF EXPR SS^c^	11.4 (2.8)	18.3 (3.4)	39.2^***^	7.2 (1.6)	18.3 (3.5)	124.0^***^
WISC NVIQ SS^d^	91.1 (19.6)	96.1 (14.5)	0.7	87.8 (14.1)	95.7 (14.9)	2.2
Ravens	95.3 (14.1)	93.8 (10.2)	0.1	83.3 (14.7)	92.3 (8.8)	4.1
BPVS SS^e^	94.2 (9.3)	104.5 (8.6)	10.5^**^	86.6 (10.3)	104.2 (8.9)	25.4^***^
BAS reading SS^f^	101.8 (12.3)	104.8 (10.5)	0.6	73.9 (9.7)	104.5 (10.8)	67.2^***^
TOWRE SS^g^	97.3 (17.0)	102.1 (9.4)	1.0	69.5 (11.0)	101.1 (8.7)	75.9^***^
BAS spelling SS	101.2 (17.6)	106.3 (12.7)	0.9	68.4 (8.9)	104.3 (8.9)	87.6^***^
Oddity rhyme (out of 20)	13.4 (4.3)	15.0 (3.1)	1.5	8.4 (3.0)	14.9 (3.1)	33.3^***^
PSTM^h^ (words correct)	36.3 (13.5)	41.8 (8.0)	1.9	30.4 (8.1)	42.4 (7.8)	17.1^***^
RAN^j^(seconds)	45.7 (24.4)	36.4 (7.0)	2.2	55.4 (18.2)	35.7 (6.3)	15.8^***^

Standard deviations in parentheses. ^*^p < 0.05,

** *p < 0.01*,

*** *p < 0.001*.

a *SS, standard score*.

b *Clinical Evaluation of Language Fundamentals, Receptive*.

c *Clinical Evaluation of Language Fundamentals, Expressive*.

d *WISC non-verbal IQ*.

e *British Picture Vocabulary Scales*.

f *British Ability Scales single word reading*.

g *Test of Word Reading Efficiency combined score*.

h *Phonological short-term memory*.

j *Rapid Automatised Naming combined score*.

### Standardized tests

Language abilities were measured through the use of two receptive subtests (Concepts and Directions, and Semantic Relations or Sentence Structure, depending on the child's age) and two expressive subtests (Formulating Sentences, and Sentence Assembly or Word Structure, depending on the child's age) of the CELF-3 (Semel et al., 1995). For all children, receptive vocabulary was measured through use of the BPVS, and single word reading was assessed using the BAS and TOWRE tests. All children also completed four subscales of the WISC III: Block Design, Picture Arrangement, Similarities, and Vocabulary. These four scales yield an estimate of full-scale IQ (pro-rated, see Sattler, 1982), and the two non-verbal scales (Block Design, Picture Arrangement) were used to gain an estimate of non-verbal IQ following the procedure adopted by Sattler (1982, p.166-7). Non-verbal IQ was also assessed using the Ravens. There were no significant non-verbal IQ differences between the matched sub-groups, as shown in Table 2.

### Phonological tasks

Children with SLI who also present with poor reading would be expected to have phonological processing difficulties, whereas the Pure SLI group identified here should not show phonological difficulties. Three experimental measures of phonological processing, previously used with children with dyslexia, were therefore also administered. As shown in Table 2, the Pure SLI group did not show phonological processing difficulties in these tasks compared to control children, whereas the SLI PPR group did show phonological difficulties.

#### Rhyme oddity task

Children listened to sets of three words and had to select the nonrhyme (e.g., boot, *cool*, root; Goswami et al., 2013). The words were presented by computer through headphones using digitized recordings of speech produced by a female native speaker of Standard Southern British English, and trials were presented in one of three fixed random orders. The task comprised 20 trials. Two practice trials with feedback were given prior to the experimental trials.

#### Phonological short-term memory task

The children heard four monosyllabic consonant-vowel-consonant words presented by computer through headphones using digitized recordings of speech produced by a female native speaker of Standard Southern British English (e.g., *type, rib, nook, bud*; originally used in Thomson et al., 2005). The children were required to repeat back the words as spoken. Sixteen trials were presented in total, eight comprising items drawn from dense phonological neighborhoods, and eight trials comprising items drawn from sparse phonological neighborhoods. The total number of items reported correctly out of 64 was used in the analyses.

#### Rapid automatized naming (RAN) task

In the RAN task, children were asked to name line drawings of two sets of familiar objects (first set: *cat, shell, knob, zip, thumb*; second set: *web, fish, book, dog, cup*; see Richardson et al., 2004). For each set, children were first introduced to the names of the pictures and then shown a page with the same pictures repeated 40 times in random order. The children were asked to produce the names as quickly as possible. Average naming speed across the two lists in seconds was used in the analyses.

### Auditory tasks

A set of auditory processing tasks using non-speech stimuli (sine tones) or speech stimuli (the syllable “ba,” described further below) were created or adapted for this project by RC. Detailed information regarding group performance on these tasks is given in Cumming et al. (under submission) and is not repeated here. The stimuli were presented binaurally through headphones at 75 dB SPL. Earphone sensitivity was calculated using a Zwislocki coupler in one ear of a KEMAR manikin (Burkhard and Sachs, 1975). The tasks used a cartoon “Dinosaur” threshold estimation interface originally created by Dorothy Bishop (Oxford University). An adaptive staircase procedure (Levitt, 1971) using a combined 2-down 1-up and 3-down 1-up procedure was used, with a test run terminating after 8 response reversals or the maximum possible 40 trials. The threshold was calculated using the measures from the last four reversals. This indicated the smallest difference between stimuli at which the participant could still discriminate with a 79.4% accuracy rate. The children were assessed individually in a quiet room within their school or at home. A rigorous practice procedure (five trials) was applied prior to the presentation of the experimental stimuli. For all the Dinosaur tasks (unless otherwise stated below in the individual task descriptions), an AXB paradigm was used; three sounds were presented consecutively, as if they were the sounds made by three distinctive cartoon dinosaurs on screen (500 ms ISI). The middle stimulus (X) was always the standard stimulus and either the first (A) or the last (B) stimulus was different from the standard. At the start of each task, the child was introduced to three cartoon dinosaurs, and for each trial the child was asked to choose which dinosaur produced the target sound i.e., whether A or B was different from X. Feedback was given online throughout the course of the experiment. All the speech stimuli were based on the monosyllable [bː], and were resynthesized from a natural [bː] token produced by a female native speaker of Standard Southern British English. She was recorded in a sound-attenuated booth; the equipment used was a Tascam DR-100 handheld recorder with an AKG C1000S cardioid microphone. One [bː] token was selected for manipulation and saved in.wav format. Details of the stimulus manipulations, which were done with the software Praat (Boersma and Weenink, 2010), are given below. All “ba” tasks were run with the Dinosaur program, but the cartoon animals that appeared on screen were sheep (because sheep say “baaa”).

#### Amplitude rise time (ART) tasks

For the non-speech task, three 800 ms sinusoid tones (500 Hz) were presented. The second tone was always a standard tone (X), with a 15 ms linear ART, 735 ms steady state, and a 50 ms linear fall time. One of the other two tones was identical to this standard, and the other tone varied in linear ART. For this variable ART, a continuum of 39 stimuli was used which increased in 7.3 ms steps from the standard to the tone with the longest ART at 293 ms. It was explained that each dinosaur would make a sound and that the child's task was to decide which dinosaur made the sound that started off more quietly and got louder more slowly than the other two dinosaurs (longer ART). In previous papers by Goswami and colleagues, this task has been called the “1 Rise” task. For the *Speech* task, three [bː] stimuli with a duration of 300 ms and a flat f0 at 200 Hz were presented. The second [bː] was always a standard stimulus (X), with a 10 ms ART. One of the other two stimuli was identical to this standard, and the other stimulus varied in ART. For this variable ART, a continuum of 39 stimuli was used which increased in 3.7 ms steps from the standard to the stimulus with the longest ART at 150 ms. This continuum was created by copying the original [bː] token 39 times, and resynthesing each copy with a specified ART using the *IntensityTier* function in Praat. The standard stimulus also underwent resynthesis from the original token, but without a change of rise time. It was explained that each sheep would make a sound and the child's task was to decide which sheep didn't make a proper “b” sound at the start compared to the other two sheep (longer ART). (This instruction was decided on after pilot tests showed it was the best description and children understood what was meant as soon as they heard the practice trials).

#### Duration tasks

For the nonspeech task, three 500 Hz sinusoid tones with a 50 ms linear ART and 50 ms linear fall time were presented. The second tone was always a standard tone (X) at 125 ms (note that this is a measure of shorter durations than those used by (Corriveau et al., 2007), which varied between 400 and 600 ms). One of the other two tones was identical to this standard, and the other varied in duration. For this variable duration, a continuum of 39 stimuli was used which increased in 3.2 ms steps from the standard to the longest tone at 247 ms. It was explained that each dinosaur would make a sound and that the child's task was to decide which dinosaur made the sound that was longer. For the *Speech* task, three [bː] stimuli with a flat f0 at 200 Hz were presented. The second [bː] was always a standard stimulus (X) at 150 ms. One of the other two stimuli was identical to this standard, and the other stimulus varied in duration. For this variable duration, a continuum of 39 stimuli was used which increased in 3.9 ms steps from the standard to the longest stimulus at 300 ms. This continuum was created by copying the original [bː] token 39 times, and resynthesising each copy with a specified duration using the *DurationTier* function in Praat. The standard stimulus also underwent resynthesis from the original token, but without a change of duration. It was explained that each sheep would make a “baa” sound and that the child's task was to decide which sheep made the “baa” sound that was longer.

#### Frequency (rising f0) tasks

Three 300 ms sinusoid tones with a 5 ms linear ART and 5 ms linear fall time were presented. The second tone was always a standard tone (X) with a 10 ms fundamental frequency (f0) rise time from 295 to 500 Hz (hence dynamic f0). One of the other two tones was identical to this standard, and the other tone varied in f0 rise time. For this variable f0 rise time, a continuum of 39 stimuli was used which increased as an exponential function from the standard to the tone with the longest f0 rise time at 150 ms. It was explained that each dinosaur would make a sound and that the child's task was to decide which dinosaur made the sound that started “wobbly” compared to the other two dinosaurs (longer f0 rise time). (This instruction was decided on after pilot tests showed it was the best description and children understood what was meant as soon as they heard the practice trials). For the *Speech* task, three [bː] stimuli with a duration of 300 ms were presented. The second [bː] was always a standard stimulus (X) with a 10 ms f0 rise time from 130 to 220 Hz (hence dynamic f0). (The onset of the f0 rise was the point of vowel onset (as opposed to syllable onset), because f0 would not be perceptible during the silence of the closure and the aperiodicity of the burst releasing the plosive [b].) One of the other two stimuli was identical to this standard, and the other stimulus varied in f0 rise time. For this variable f0 rise time, a continuum of 39 stimuli was used which increased as an exponential function from the standard to the stimulus with the longest f0 rise time at 150 ms. This continuum was created by copying the original [bː] token 39 times, and resynthesizing each copy with a specified f0 rise time using the *PitchTier* function in Praat. The standard stimulus also underwent resynthesis from the original token, but without a change of f0 rise time. It was explained that each sheep would make a sound and that the child's task was to decide which sheep made the sound that started “wobbly” compared to the other two sheep (longer f0 rise time).

### Music and speech tasks

#### Beat perception in music task

**Figure 1:**
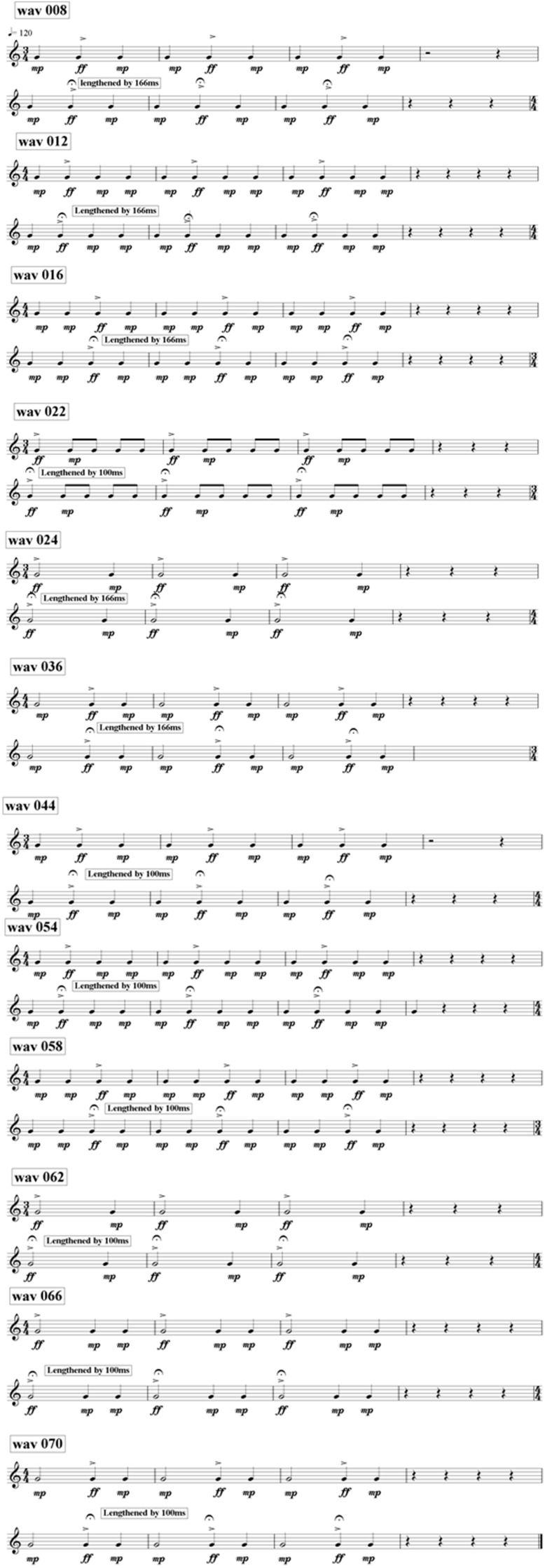
**The “tunes” in the musical beat perception task**. Reprinted from Goswami et al. (2013) with permission from Elsevier.

This was the same task used with children with dyslexia by Goswami et al. (2013). It was a shortened version of the “musical meter” task originally reported by Huss et al. (2011). The task comprised 24 trials of different beat structure arrangements of a series of notes with an underlying pulse rate of 500 ms (120 bpm). Twelve of the trials delivered the identical series of notes twice (“same” trials), and 12 delivered two slightly different series of notes (“different” trials). Different trials were created by elongating the accented note by either 100 ms or 166 ms. All of the “different” trials are provided as Figure 1. The “same” trials were the identical arrangements without a lengthening of the accented note. The sound files were originally created by John Verney at the Centre for Neuroscience in Education, University of Cambridge, using Sibelius Version 4 from a sound set produced by Native Instruments (Kontakt Gold). Hence the “tunes” sounded musical with appropriate timbre and slow decay times. Fourteen trials (7 same, 7 different) were in 4/4 time and 10 trials (5 same, 5 different) were in 3/4 time. The delay in the rhythm structure was either short (100 ms, 7 “different” trials) or long (166 ms, 5 “different” trials). The child's task in all cases was to make a same-different judgment: were the two “tunes” the same or different? Trials were delivered in a pseudo-random order. The % of trials judged correctly was the variable used for data analysis. Further details can be found in Huss et al. (2011) and Goswami et al. (2013).

#### Tapping to music task

Two short pieces of instrumental music written by John Verney of the Centre for Neuroscience in Education, Cambridge, were adapted for this study. Each piece was played to the children via headphones, each was 20 s long. One piece of music was composed in 4/4 time, and the second piece was in 3/4 time, and each piece was composed so that the underlying beat rate was 500 ms. The children were asked to tap along in time to the music using the computer mouse, which in this case was a single large button. We took great care to explain to the children and demonstrate how they should tap briefly on the button along to the beat (rather than pressing and holding, which could lead to beat sub-division). This was expected to result in a tap rate (Tactus) of around 500 ms, however some children tapped once every two beats (1000 ms). The tapping was recorded by specially-written software so that inter-tap intervals could be analyzed.

To score performance for each participant, we report two methods. One is a synchronization measure that is derived from the log-transform of Absolute Error (Spray, 1986), a measure of the accuracy of motor performance. The synchronization score is an unambiguous measure of the child's accuracy in tapping at the tactus rate. The second measure represented individual differences in the children's performance using circular statistics, which are increasingly popular in motor synchronization studies (Sowinski and Dalla Bella, 2013; Falk et al., 2015). Circular statistics are most useful when there is an unambiguous pacing signal, for example a metronome beat. As our pieces of music did not include a pacing signal of this nature, we utilized the temporal offset of the dominant percussion instrument to compute circular statistics, as detailed below.

##### Synchronization score (sync score)

Before data processing the first and last tap were removed from the sequence (we did not remove more taps as the sequences were only 20 s long). To compute the synchronization score, the absolute distance (in ms) from the “ideal” tap rate was computed for each child, at his/her chosen tactus level (which could be either 500 ms or 1000 ms, depending on whether children tapped on every beat or on every two beats). Therefore, this distance was |(median ITI – 500)|for the 500 ms tactus, and |(median ITI – 1000)|for the 1000 ms tactus. The absolute value was used because children who tapped at −100 ms from the ideal rate were deemed to be as accurate as those who tapped at +100 ms from the ideal rate. Median scores were used rather than mean scores to minimize the effects of breaks in tapping (when children missed out a beat). Group variability in tapping is captured by the variance and standard deviations of these median scores. As the resulting absolute values could not be negative, resulting in a heavily skewed distribution, a logarithmic transform was applied. A negative log was used so that large distances would result in small scores and vice versa. “+1” was added to each score *before* the logarithmic transform to prevent infinite values if participants were perfectly synchronized (i.e., log 0). Finally, “+6” was added to the score *after* the logarithmic transform so that all resulting scores would be positive. Therefore, final synchronization scores could range in value between 0 (very poor) to 6 (perfect; in fact they ranged between 0.35 and 6). The formula for the SyncScore is shown in Equation (1) below:

(1) $$\begin{array}{l} \text{SyncScore}=\left(-\text{log}\left(|\text{ITI-tactus}|+1\right)+6\right) \end{array}$$

These synchronization scores were then used in further data analysis.

##### Circular statistics

Before calculating the circular metrics for a trial, the first and last taps were removed. Any taps with an offset deviating from the mean by more than two standard deviations were also removed Circular statistics transform the asynchronies between the tapping and pacing signal into a point on the circumference of a unit circle. Taps that are aligned perfectly are placed at 0 radians on the circle, while taps that fall exactly between two beats of the pacing signal are placed at π/2 radians. Consideration of all the angles obtained in a particular trial enables calculation of a vector corresponding to the mean angle. The direction of this vector gives a measure of the degree to which the taps deviate, on average, from the pacing signal at 0′. The length of the vector gives a measure of the variability of phase alignment or phase locking strength. The direction is sometimes interpreted as a measure of synchronization *accuracy* (e.g., Falk et al., 2015). For the purposes of the present study, however, this is not the case. For example, if one group of children were tapping in exact anti-phase to the pacing signal, this would not necessarily signify low accuracy, as the children may still be responding by producing taps with spacing that corresponds to the beat of the music (the tactus). Consequently, in the current study vector length most closely corresponds to synchronization accuracy. For example, a child who taps reliably out of phase but at the exact tactus of the music will have a high mean angle and a vector length close to 1. However, a child who has taps that vary in ISI from below to above the tactus may have a mean angle of close to 0, but a much smaller vector length. The participant in the first case would also obtain a high SyncScore, while the participant in the second case would obtain a low SyncScore. Although we report both mean angle and vector length below, the data that we obtain from applying circular statistics should be interpreted with this in mind.

#### Pitch and rhythm in music and speech (music/speech AXB tasks)

Six tasks varying rhythm and/or pitch were created by RC by adapting a listening game based on Pingu (a cartoon penguin whose speech was difficult to understand) originally developed by Richards (2010). There were three musical tasks and three speech tasks, all of which used an AXB paradigm. In each case, one of the comparison stimuli (A or B) had the same rhythm and/or pitch contour as the standard (X), while the other did not. The children thus had to match the comparison stimuli with the standard on the basis of shared rhythm, shared pitch, or both. The music tasks were created by low-pass filtering a short tune. Children were shown a picture of a radio, and listened to the tune (standard stimulus: X). They then saw two pictures, one of a girl dressed in green playing the piano and one of a girl dressed in blue playing the piano. Two short unfiltered piano tunes were played (comparison stimuli: A—from the girl in green; B—from the girl in blue). Finally they heard the filtered radio tune again. The children were asked whether it was the same as that played by the girl in green or the girl in blue. For the speech tasks, the children saw a picture of a door, and a “muffled” voice spoke a sentence as if from behind the door (standard stimulus: X). The children were then shown two pictures, one of a lady dressed in green and one of a lady dressed in purple, and heard two sentences of natural (unfiltered) speech (comparison stimuli: A—from the lady in green; B—from the lady in purple). Finally they heard the muffled sentence again. The children were asked whether it was the same as that said by the lady in green or by the lady in purple. An overview of the tasks is given in Table 3.

**Table 3:** **Details of the novel Music/Speech AXB tasks**.

**Modality**	**Disrupted**	**Standard stimulus X (Music or Speech)**		**Comparison stimuli (A,B)**
		**Filtered: “on the radio” or “behind the door”**		**Both unfiltered**
		**Rhythm (durational properties and amplitude modulations)**	**Pitch (f0) contour**	
Music	Rhythm only	Pattern of “strong” (longer, louder) and “weak” (shorter, quieter) notes e.g., SSWSS	Flat: one note (A) at 441 Hz	Only one matches X in rhythm
	Pitch only	Series of notes with identical duration and amplitude	Melodious: various notes	Only one matches X in f0 contour
	Rhythm and pitch	Pattern of “strong” (longer, louder) and “weak” (shorter, quieter) notes e.g., SSWSS	Melodious: various notes	Both have melodious pitch contours, but only one matches X in rhythm
Speech	Rhythm only	Syllable string with certain pattern of durational properties and amplitude modulations	Flat: 223 Hz	Only one matches X in rhythm
	Pitch only	Syllable string comprising fully sonorant sounds: no breaks in periodicity, and relatively little amplitude information (excepting the nuclear-accented syllable)	Melodious: intonation pattern with rises and falls	Only one matches X in f0 contour
	Rhythm and pitch	Syllable string with pattern of durational properties and amplitude modulations	Melodious: intonation pattern with rises and falls	Both have melodious pitch contours, but only one matches X in rhythm

##### Music tasks

The stimuli were melodies (5–7 notes) played by a single piano, created using the software Sibelius (version 6) from a sound set produced by Native Instruments (Kontakt Gold). A copy of each tune (.wav format) underwent filtering with a 0–500 Hz pass band and 50 Hz smoothing, using the software Praat (Boersma and Weenink, 2010). This left enough spectral information to hear the durational properties and the amplitude and fundamental frequency (f0) modulations, but the melodies no longer sounded like a piano (which fitted the picture story, that the radio played a poorer quality sound than a live performance). Each filtered tune became a standard stimulus (X), and the natural (unfiltered) tunes became the comparison stimuli (A, B).

##### Music: rhythm only

In this task, only rhythm was disrupted, hence both comparison stimuli had the same f0 contour as the standard. This meant that successful matching to the standard depended on matching rhythm. To disrupt the rhythm, the standard rhythm pattern of “strong” and “weak” notes was altered by changing a strong note for a weak note or vice versa (see Table 3). The strong notes had a longer duration (crotchet or quarter note) than the weak notes (quaver or eighth note), and a higher amplitude (specified as fortissimo in Sibelius) than the weak notes (no dynamic indication in Sibelius). The location of this exchange of notes (i.e., beginning/middle/end of the tune) varied between trials.

##### Music: pitch only

In this task, only pitch was disrupted. This meant that successful matching to the standard depended on matching pitch contour. The tunes comprised notes in the same rhythm (no strong-weak pattern), but with varying melodies (i.e., different f0 contours). For each trial, the two comparison stimuli differed by the exchange of two notes with a different f0, e.g., E B A A B compared to E B A B A—here the 4th and 5th notes were swapped. The location of this exchange of notes (i.e., beginning/middle/end of the tune) varied between trials. There were seven trials in which the f0 contour across the two notes involved in the exchange was falling in stimulus A and rising in stimulus B, and seven trials in which the f0 contour across the two notes was rising in A and falling in B.

##### Music—rhythm and pitch

In this task, tunes comprised notes with varying rhythms and melodies. For each trial, the two comparison stimuli differed by the exchange of a strong note for a weak note or vice versa, e.g., SSWSS compared to SSWSW. Both comparison stimuli in each trial had the same melody, however this exchange meant that the rhythm differed between the comparison stimuli and the f0 contour differed within each stimulus. The effect was that the discrepant tune sounded different in both rhythm and pitch, which should make selection of the correct match easier. A fuller description of each task is provided in the Appendix of Supplementary Material.

##### Speech tasks

The stimuli were sentences (5–7 syllables) appropriate for primary school children in terms of their syntactic and lexical complexity. A female speaker of Standard Southern British English was recorded in a sound-attenuated booth producing each sentence 5 times; the equipment used was a Tascam DR-100 handheld recorder with an AKG C1000S cardioid microphone. The token of each sentence with the best fluency and stress pattern was selected to make the stimuli. This token (.wav format) underwent filtering with a 0–500 Hz pass band and 50 Hz smoothing, using the software Praat (Boersma and Weenink, 2010). Following filtering, enough spectral information remained to hear the durational properties and amplitude and f0 modulations (i.e., prosody), but it was no longer possible to decipher what was said (which fitted the idea of speech being muffled by the door). Each filtered sentence became a standard stimulus (X), and the natural (unfiltered) sentences became the comparison stimuli (A, B).

##### Speech—rhythm only

In this task, only speech rhythm was disrupted, as both comparison stimuli had the same (flat) f0 contour. This meant that successful matching to the standard depended on matching rhythm. The stimuli hence comprised three sentences with the same flat f0, with varying rhythm. The f0 was manipulated in Praat, by making a copy of each original sentence and resynthesising it with a flat f0 at 223 Hz (the mean f0 of all the original sentence recordings). The standard stimulus (X, filtered speech) for each sentence was also manipulated to have a flat f0 at 223 Hz using the same method. For each trial, the comparison stimuli comprised different words, but had the same number of syllables and were not perceptibly different to X in overall duration. The difference in rhythm was created by manipulating the syllable upon which the nuclear accent (or main sentence stress) fell. This nuclear-accented syllable had a perceptibly higher amplitude and longer duration, accounting for inherent phoneme length, than surrounding syllables (the large f0 excursion of the nuclear accent was lost once the f0 was flattened). The difference in the words used also contributed to the rhythmic difference, because the duration and amplitude of each individual syllable partly determined the rhythm too. (For more details, see Appendix in Supplementary Material).

##### Speech—pitch only

In this task, only pitch contour was disrupted and all sentences had the same rhythm. Sentences were created using words with only fully sonorant sounds. Therefore, in the filtered (X) stimuli: (a) there were no breaks in periodicity, so no syllable boundaries were detectable, hence there was no durational information for individual syllables; and (b) the filtered sonorant sounds all had a similar amplitude, hence relatively little amplitude information for individual syllables was available (except the nuclear-accented syllable). For each trial, the comparison stimuli used different words, but had the same number of syllables as X and were not perceptibly different in overall duration. The difference in pitch (f0 contour) was again created by varying which syllable received the nuclear accent. This nuclear-accented syllable had a large f0 excursion, as well as a perceptibly higher amplitude than surrounding syllables. So the primary difference between the standard and comparison sentences was carried by pitch contour, even though both f0 and amplitude modulations were varied (although durational properties were kept constant). Note that this latter variability was unavoidable, as speech is a more complex signal than a piano tune, and therefore controlling acoustic variables whilst keeping the stimuli natural-sounding is challenging. Nevertheless, as there were no detectable syllable boundaries, it was not possible to perceive amplitude modulations relative to individual syllable durations. Therefore, the correct choice was necessarily based on recognizing an overall contour of f0 (and amplitude) modulation across the standard and comparison sentences (for more details of all trials, see Appendix in Supplementary Material).

##### Speech—rhythm and pitch

In this task, both pitch and rhythm were disrupted, which should make correct matching easier. The stimuli comprised sentences with various rhythms and modulated f0 contours. For each trial, the comparison stimuli used different words, but had the same number of syllables and overall duration. The difference in rhythm and f0 contour was again created by letting the nuclear accent fall on different syllables. The nuclear-accented syllable had a perceptibly higher amplitude and longer duration (accounting for inherent phoneme length) than surrounding syllables, and a large f0 excursion. As the words in the standard and comparison sentences also differed, there was also a rhythmic difference. (For further details, see Appendix in Supplementary Material).

#### Scoring

The tasks were presented in PowerPoint. For each condition, there were 14 trials preceded by two practice trials, presented in a fixed random order. The correct match to stimulus X was A in half of the trials and B in half of the trials. Before the first trial, the child viewed the pictures with the experimenter, who explained the task without the sounds. For each experimental trial, once the stimuli had played through in sequence (X-A-B-X, ISI 1 s), the child was given an unlimited response time. Children could ask for the trial to be repeated if necessary. Responses were scored as three points for each correct answer after one listening, two points after two listenings, one point after three or more listenings, and 0 points for each incorrect answer. This gave a maximum score out of 42 for each condition, with 42 representing ceiling performance (every answer correct after listening once). The children completed all six tasks in one of two fixed orders, with three tasks given in one test session and three given in a separate session. Each task took between 7 and 10 min to complete.

## Results

Group data for the musical beat perception and tapping tasks and the speech/music AXB tasks are shown in Table 4, which presents group average performance for the three groupings of SLI children (Pure SLI, SLI PPR, whole group with IQ varying) and their TD controls. ANOVA was used as the main analysis technique, although note that as the TD controls for the Pure SLI grouping and the SLI PPR grouping were partly similar and partly different, we could not incorporate all three groups into one ANOVA (Pure SLI, SLI PPR, TD) as this removed the IQ-matching. Further, as there is no straightforward circular equivalent of the mixed-effects ANOVA, we used *t*-tests when analyzing children's synchronization to the musical beat via circular measures.

**Table 4:** **Performance in the music and speech tasks by group**.

	**All SLI *N* = 45**	**All controls *N* = 50**	**Pure SLI *N* = 16**	**Pure controls *N* = 16**	**SLI PPR *N* = 15**	**PPR controls *N* = 15**
Musical beat perception, % corr	61.3 (14.7)	70.7 (14.9)	64.9 (13.5)	70.6 (12.3)	63.9 (16.4)	71.5 (12.2)
Speech, match on rhythm^a^	25.0 (7.2)	30.5 (7.3)	27.4 (7.9)	28.9 (8.1)	22.5 (7.3)	29.0 (8.4)
Speech, match on rhythm, and pitch^a^	27.0 (8.9)	34.8 (8.5)	29.7 (8.8)	32.9 (9.2)	28.7 (8.7)	34.1 (8.2)
Speech, match on pitch^a^	24.1 (7.8)	26.2 (9.0)	25.5 (10.2)	24.9 (9.0)	25.6 (9.0)	24.9 (6.4)
Music, match on rhythm^a^	25.2 (7.6)	33.4 (6.6)	26.6 (8.7)	34.2 (6.4)	24.5 (8.5)	34.4 (6.6)
Music, match on rhythm and pitch^a^	25.9 (7.6)	35.1 (6.9)	31.0 (8.8)	31.9 (7.8)	24.3 (6.0)	32.8 (7.2)
Music, match on pitch^a^	24.2 (6.6)	30.6 (5.7)	26.9 (7.0)	30.3 (5.1)	23.2 (5.3)	30.7 (4.9)
Tapping, 3/4 time (sync score)	2.3 (1.7)	3.2 (1.5)	2.0 (1.5)	3.0 (1.3)	2.6 (1.9)	3.0 (1.1)
Tapping, 4/4 time (sync score)	1.8 (1.4)	2.6 (1.7)	1.8 (1.5)	2.5 (1.6)	1.8 (1.6)	2.3 (1.4)
Tapping 3/4 time angle	−0.5 (0.23)	−0.5 (0.21)	−0.41 (0.15)	−0.42 (0.19)	−0.56 (0.22)	−0.44 (0.19)
Tapping 4/4 time angle	−0.45 (0.14)	−0.52 (0.23)	−0.44 (0.19)	−0.51 (0.22)	−0.47 (0.13)	−0.51 (0.23)
Tapping 3/4 time vector length	0.97 (0.03)	0.97 (0.03)	0.96 (0.03)	0.98 (0.02)	0.97 (0.02)	0.98 (0.02)
Tapping 4/4 time vector length	0.97 (0.02)	0.97 (0.03)	0.96 (0.03)	0.98 (0.02)	0.97 (0.02)	0.98 (0.02)

*Standard deviations in parentheses*.

a *Maximum score = 42*.

### Musical beat perception

Musical beat perception was significantly above chance (50%) for all groups. For the Pure SLI group and their TD controls, a One-way ANOVA by group showed no difference in performance [64.9% correct vs. 70.6% correct, *F*_(1, 30)_ = 1.6, *p* = 0.22]. There was also no significant difference in musical beat perception for the SLI PPR children and their TD controls, *F*_(1, 28)_ = 2.0, *p* = 0.17 (mean scores 63.9% correct vs. 71.5% correct). There was a significant group difference for the whole SLI sample with IQ varying, however, as performance was significantly better for the control children (70.7% correct) than the SLI children [61.3% correct; *F*_(1, 94)_ = 9.7, *p* = 0.002]. Hence sensitivity to patterns of beats in music as measured by this task appears to be preserved in SLI children with intact NVIQ.

### Tapping to music

One child was absent for the tapping tasks (CA control). For the Pure SLI children, a 2 × 2 [Group × Tempo (3/4 time, 4/4 time)] ANOVA taking the SyncScore as the dependent variable showed a significant main effect of Group only, *F*_(1, 29)_ = 5.3, *p* = 0.03. There was no interaction between Tempo and Group, *F*_(1, 29)_ = 0.5, *p* = 0.47, and no main effect of Tempo, *F*_(1, 29)_ = 1.2, *p* = 0.29. Hence the children with Pure SLI showed significantly poorer motor synchronization with the beat compared to the TD controls. For the SLI PPR children, a second 2 × 2 ANOVA utilizing the SyncScores showed a significant main effect of Tempo, *F*_(1, 27)_ = 4.5, *p* = 0.04, because synchronizing with the beat in 4/4 time was easier for all children. The main effect of Group approached significance, *F*_(1, 27)_ = 3.0, *p* = 0.09, and there was no interaction between Tempo and Group, *F*_(1, 27)_ = 0.7, *p* = 0.79. For the whole sample, the SyncScore ANOVA showed a significant main effect of Tempo, *F*_(1, 92)_ = 7.5, *p* = 0.007, because music in 4/4 time was significantly easier to synchronize to. The main effect of Group was also significant, *F*_(1, 92)_ = 9.7, *p* = 0.002, but there was no interaction between Tempo and Group, *F*_(1, 92)_ = 0.08, *p* = 0.78. Hence overall the SLI children showed less synchronization to the beat than the TD control children.

A second set of analyses was also conducted using circular statistics. Circular statistics produce two metrics that can be submitted to data analysis: mean angle and vector length. Before submitting vector length to analysis the data were first logit transformed because these data were heavily skewed. Furthermore, because no straightforward way exists to conduct mixed ANOVA on circular data, separate analyses were conducted at each level of the within-subject factor (Tempo: 4/4 time, 3/4 time) for both metrics (vector length and vector angle; note that conceptually vector length is expected to be the variable that mirrors performance as characterized by the SyncScores). For the Pure SLI children and vector length, *t*-tests revealed no significant differences between the tapping behavior of the SLI children and the TD controls for the 3/4 tempo, *t*_(29)_ = 1.487, *p* = 0.148. For the 4/4 tempo, however, SLI children tapped less consistently than their TD controls, *t*_(28)_ = 2.483, *p* = 0.019. These results are consistent with the results obtained using the SyncScore. For the SLI PPR children and vector length, no differences were found in their tapping behavior relative to controls for the 3/4 tempo, *t*_(27)_ = 0.554, *p* = 0.584, nor for the 4/4 tempo, *t*_(27)_ = 1.596, *p* = 0.122. These results again match those obtained with the SyncScore. For the whole sample, no differences were found between the tapping behavior of the SLI children and the TD control group using the vector length measure, for both the 3/4 tempo, *t*_(91)_ = 1.68, *p* = 0.096, and the 4/4 tempo, *t*_(91)_ = 1.6, *p* = 0.113. Hence for the whole group of SLI children (IQ varying), the vector length measure did not show the significant group differences revealed by the SyncScore measure, however the Group effect did approach significance for 3/4 time.

The final circular statistical analysis was conducted on mean angle or direction. Data were analyzed separately for each tempo using a Watson-Williams test (implemented in the MATLAB circular statistics toolbox). The Watson-Williams tests revealed no significant differences in synchronization between SLI and TD controls for either the full sample or the subsamples (Pure SLI, SLI PPR), for both tempi (3/4 time, 4/4 time; all *p*'s > 0.50). We also explored the Spearman correlation between the SyncScore and both vector length and vector angle. Vector length was significantly correlated with the SyncScore for both the 3/4 tempo, ρ = 0.247, *p* = 0.017, and the 4/4 tempo, ρ = 0.403, *p* < 0.001. Vector angle, however, was *not* correlated with the SyncScore for either the 3/4 tempo, ρ = −0.073, *p* = 0.486, or the 4/4 tempo, ρ = 0.027, *p* = 0.800. Because the analyses using vector length and the SyncScore are largely in agreement, further analyses of tapping to music will focus on the SyncScore and vector length measures only.

### Music/speech AXB tasks

**Figure 2:**
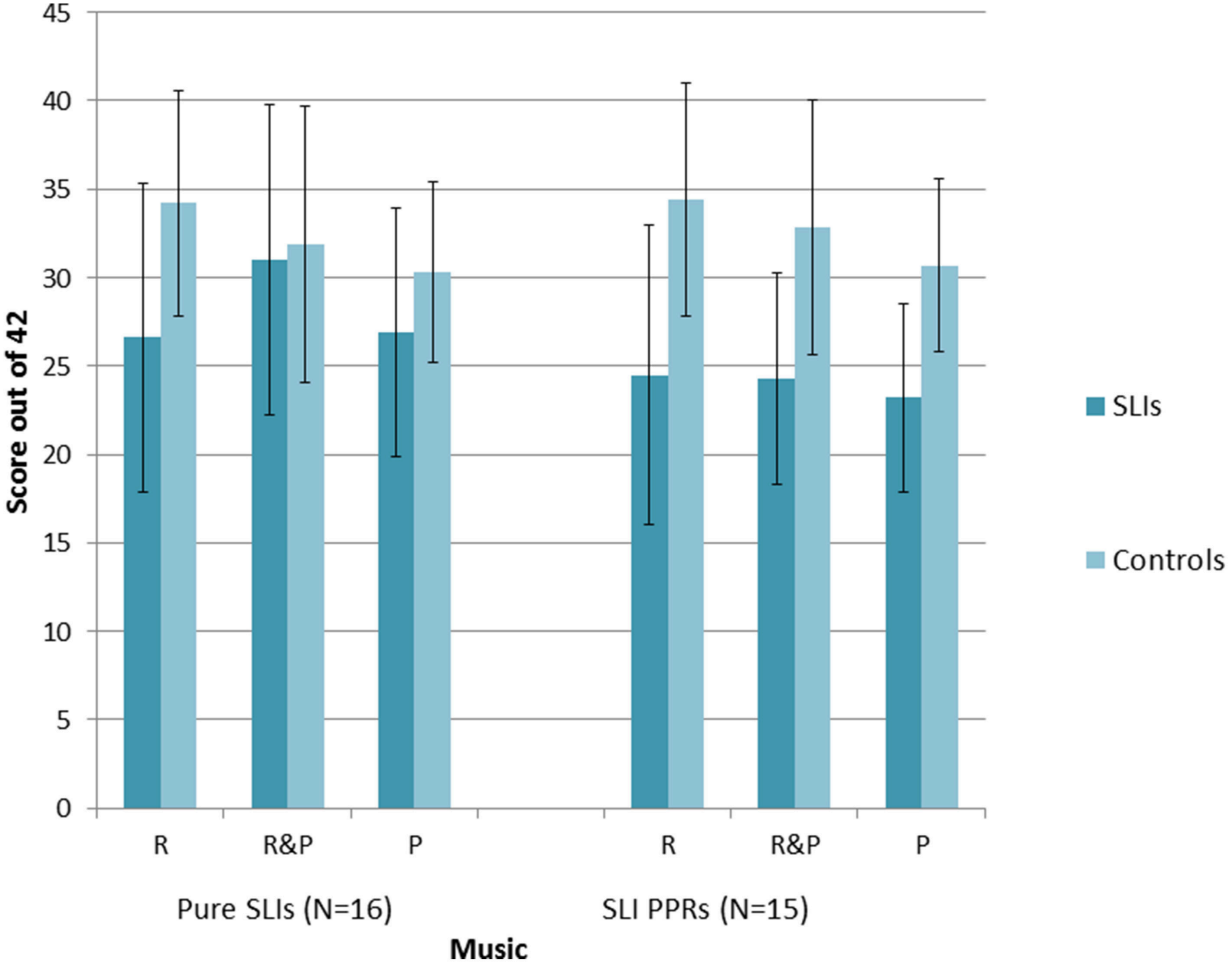
**Comparison of performance in the Music AXB tasks: Pure SLI vs. SLI PPR Groups**. The bars denote the standard error of the mean.

**Figure 3:**
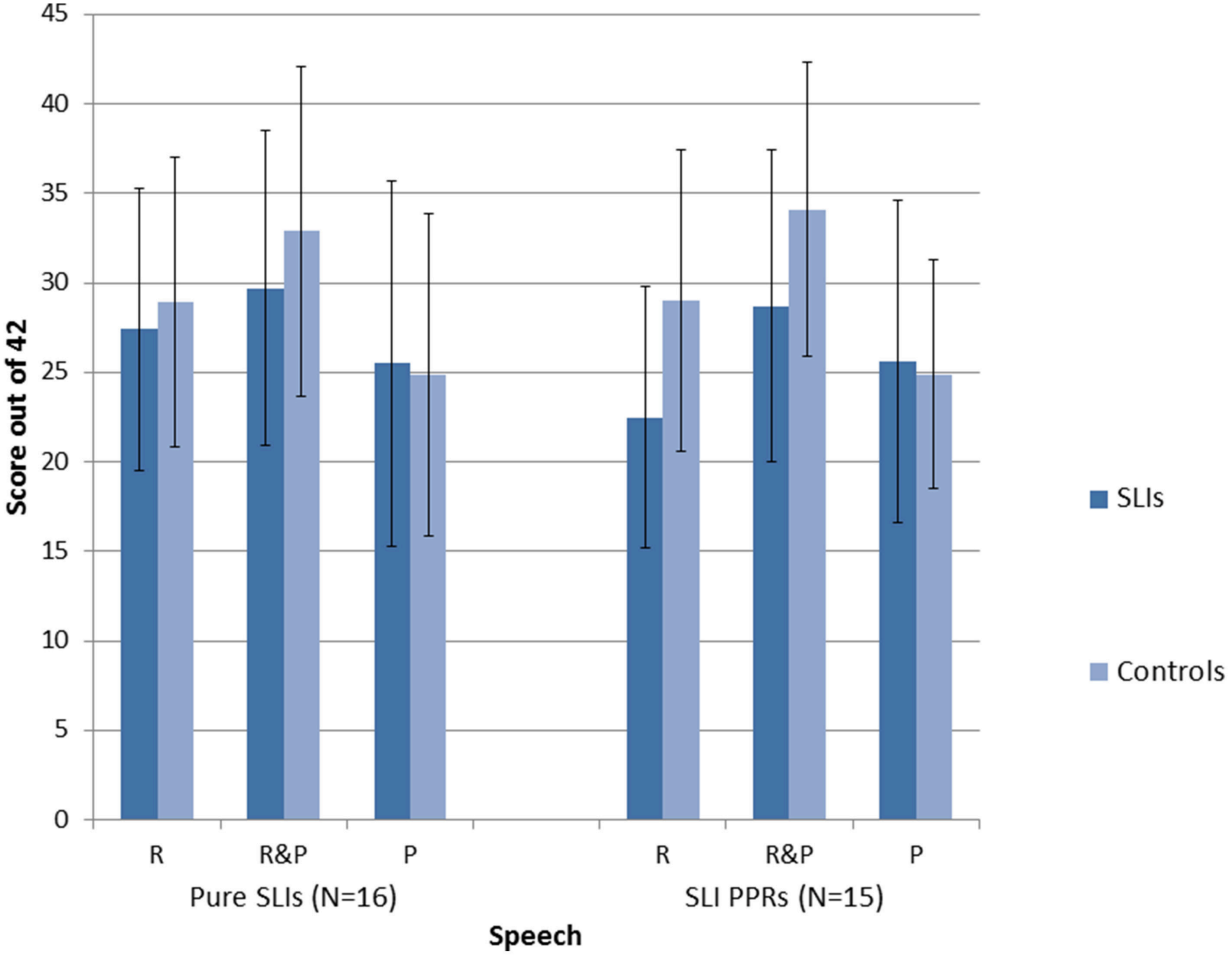
**Comparison of performance in the Speech AXB tasks: Pure SLI vs. SLI PPR Groups**. The bars denote the standard error of the mean.

A series of repeated measures 2 × 2 × 3 ANOVAs [Group × Condition (Music, Speech) × Task (Match Pitch, Match Rhythm, Match Rhythm and Pitch)] were run, one for each SLI grouping. For the Pure SLI group, the ANOVA showed a significant main effect of Condition, *F*_(1, 30)_ = 5.7, *p* = 0.006 only. Neither Group, *F*_(1, 30)_ = 2.1, *p* = 0.16, nor Task, *F*_(1, 30)_ = 3.2, *p* = 0.08, were significant, and there were no significant interactions. *Post-hoc* tests (Tukey) showed that all the children found it significantly easier to match on the basis of both Rhythm and Pitch than to match on the basis of just Pitch, *p* = 0.003. There was no significant difference between matching on the basis of Rhythm only vs. Pitch only, nor between matching on the basis of both Rhythm and Pitch vs. Rhythm only. So sensitivity to rhythm was supporting all children's performance in both the speech and music tasks. The significant trend for Task arose because the music conditions were easier than the speech conditions, *p* = 0.08. For the SLI PPR children, the ANOVA showed a significant main effect of Condition, *F*_(2, 56)_ = 5.4, *p* = 0.007, a significant main effect of Group, *F*_(1, 28)_ = 15.2, *p* = 0.001, and a significant interaction between Task and Condition, *F*_(2, 56)_ = 6.5, *p* = 0.003. *Post-hoc* testing (Tukeys) of the interaction showed that for the Speech AXB task, the children found it significantly easier to match on the basis of both Rhythm and Pitch than to match on the basis of either cue alone (both *p*'s = 0.001). The ease of matching on the basis of Rhythm alone or Pitch alone did not differ. For Music, by contrast, there were no significant differences between the three conditions. However, it was significantly more difficult for the children to match on Pitch alone in the Speech AXB task than to match on the basis of Rhythm alone in the Music AXB task, *p* = 0.003. The main effect of Group arose because the children with SLI showed significantly poorer performance than the TD children, as expected. The main effect of Task did not approach significance, *F*_(1, 28)_ = 0.5, *p* = 0.48, but the interaction between Task and Group did approach significance, *F*_(2, 28)_ = 3.5, *p* = 0.07. *Post-hoc* inspection of the interaction showed that it arose because the TD controls performed at a significantly higher level in each task than the SLI PPR children, with larger group differences for the music tasks (Music, *p* < 0.001; Speech, *p* = 0.03). The overall patterns of performance for the two groupings of SLI children with intact IQ are shown in Figures 2, 3 for the Music and Speech AXB tasks respectively. As the Figures show, the SLI PPR children tended to perform more poorly than the Pure SLI children, in the Music tasks in particular. The Music tasks also tended to be performed better by the TD children than by the SLI children.

For the whole sample with IQ varying, the 2 × 2 × 3 ANOVA showed significant main effects of Condition, *F*_(2, 186)_ = 22.4, *p* < 0.001, and Group, *F*_(1, 93)_ = 46.2, *p* < 0.001, while the main effect of Task approached significance, *F*_(1, 93)_ = 3.7, *p* = 0.06. There were also significant interactions between Group and Condition, *F*_(1, 186)_ = 5.3, *p* = 0.006, and Group and Task, *F*_(1, 93)_ = 5.5, *p* = 0.02. The main effect of Task approached significance because overall the Music tasks were easier than the Speech tasks (*p* = 0.06). *Post-hoc* inspection of the significant interaction with Group (Tukeys) showed that this was only the case for the TD children however, *p* < 0.001. The SLI children performed at similar levels in both tasks. The main effect of Condition arose because the Rhythm and Pitch conditions were significantly easier than the Rhythm only conditions, which in turn were significantly easier than the Pitch only conditions (*p*'s < 0.001). The interaction between Condition and Group arose because this effect was again due to the TD children only. The children with SLIs found all three conditions equally difficult. As would be expected, the TD children were also significantly better than the SLI children in every condition (*p*'s < 0.001).

Overall, the AXB data suggest that even children with SLI who have intact IQ find it difficult to perceive rhythm patterns in music, but that this difficulty is greater for the SLI PPR children than for the Pure SLI children. Both SLI groups with preserved NVIQ tended to find the Musical AXB tasks more difficult than the Speech AXB tasks (see Figures 2, 3). This could be due to less familiarity in listening to music vs. speech. In order to see whether individual differences in the musical beat perception, tapping-to-music and Speech/Music AXB tasks would show the expected relations with individual differences in basic auditory processing (auditory thresholds in the ART, duration and dynamic f0 tasks described earlier) rank order correlations were computed and 1-tailed tests were applied. The correlation matrix is shown as Table 5.

**Table 5:** **Spearman's rank correlations between auditory processing thresholds, tapping and performance in the music and speech tasks**.

	**Ba duration**	**Ba F0**	**Ba ART**	**Tone duration**	**Tone F0**	**Tone ART**	**Tapping 3/4 time**	**Tapping VL 3/4 time**	**Tapping SyncS SyncS 4/4 time**	**Tapping VL 4/4 time**
Musical beat perception	−0.48^***^	−0.43^***^	−0.48^***^	−0.47^***^	−0.32^**^	−0.44^***^	0.19^*^	0.31^**^	0.09	0.25^**^
Speech, match rhythm only	−0.25^**^	−0.14	−0.32^**^	−0.18^*^	−0.27^**^	−0.15	0.19^*^	0.18^**^	0.12	0.23^*^
Speech, match rhythm, and pitch	−0.30^**^	−0.28^**^	−0.34^**^	−0.41^***^	−0.43^***^	−0.25^**^	0.22^*^	0.14	0.30^**^	0.17
Speech, match pitch only	−0.12	−0.23^*^	−0.07	−0.16	−0.23^*^	−0.15	−0.08	0.11	0.09	0.15
Music, match rhythm only	−0.38^***^	−0.37^***^	−0.37^***^	−0.39^***^	−0.40^***^	−0.43^***^	0.14	0.16	0.26^**^	0.21^*^
Music, match rhythm and pitch	−0.22^*^	−0.28^**^	−0.37^***^	−0.42^***^	−0.46^***^	−0.33^**^	0.22^*^	0.00	0.26^**^	0.09
Music, match pitch only	−0.30^**^	−0.20^*^	−0.30^**^	−0.35^**^	−0.39^***^	−0.31^**^	0.18^*^	−0.06	0.25^**^	0.08
Tapping, 3/4 time	−0.06	−0.01	−0.22^*^	−0.15	−0.10	−0.23^*^		0.25^**^	0.43^***^	0.23^*^
Vector length, 3/4 time	−0.12	−0.33^***^	−0.11	−0.11	−0.16	−0.19^*^	0.25^**^		0.09	0.27^**^
Tapping, 4/4 time	−0.05	−0.09	−0.04	−0.09	−0.21^*^	−0.12	0.43^***^	0.09		0.40^***^
Vector length, 4/4 time	−0.07	−0.18^*^	−0.08	−0.10	−0.08	−0.24^*^	0.23^*^	0.27^**^	0.40^***^	

* *p < 0.05*,

** *p < 0.01*,

*** *p < 0.001*.

Inspection of Table 5 shows that most of the tasks showed significant negative correlations with the auditory processing measures (higher auditory thresholds were related to poorer task performance, as would be expected). For the Speech AXB tasks, there are some theoretically interesting exceptions. Sensitivity to ART in speech is unrelated to matching speech patterns on the basis of pitch contour, while sensitivity to rising F0 in speech is unrelated to matching speech patterns on the basis of rhythm, suggesting a cognitive dissociation. The tapping to music tasks are also unrelated to matching speech patterns on the basis of pitch contour, for both the SyncScore and vector length measures, again suggesting a dissociation between rhythmic performance and sensitivity to pitch contours. Whereas, the SyncScore accuracy measures are usually significantly related to AXB rhythm matching, for both speech and music, the vector length measures are usually not. The tapping to music measures also show relatively few significant correlations with the auditory tasks, apart from with tone ART (and vector length) and speech F0 (and both SyncScore and vector length). As noted, the mean angle data are not shown in the table, however here no correlations with the auditory tasks reached significance except for one, between Ba ART and 3/4 time, *r* = 0.24, *p* < 0.05. Regarding correlations between the different Music/Speech AXB tasks (also not shown in the table), performance in all except two of the tasks was significantly related. No relationship was found for matching rhythm in speech and matching pitch in speech (the AXB “behind the door” tasks, *r* = 0.07). This interesting pattern supports the pattern of correlations discussed above in suggesting that the “behind the door” task (using filtered speech) is successfully distinguishing children's sensitivity to pitch contours in speech from their sensitivity to durational and rise time (rhythm-related) cues.

In a second set of correlations, potential associations between performance in the music and speech tasks and performance in the standardized measures of language, phonology and reading were explored. This time positive correlations were predicted, as better sensitivity in the rhythm and pitch measures should theoretically lead to better language, reading and phonology outcomes. The correlation matrix is shown as Table 6. As expected, the matrix shows significant positive associations between performance in virtually all of the tasks. Again, no significant correlations were found for the mean angle measures, which are not shown in the table.

**Table 6:** **Spearman's rank correlations between performance in the music and speech tasks and language skills**.

	**CELF receptive**	**CELF express**	**BPVS**	**BAS read**	**BAS spell**	**TOWRE**	**Rhyme oddity**
Musical beat perception	0.38^***^	0.39^***^	0.35^***^	0.43^***^	0.36^***^	0.42^***^	0.49^***^
Speech, match rhythm only	0.35^***^	0.33^**^	0.32^**^	0.41^***^	0.42^***^	0.41^***^	0.42^***^
Speech, match rhythm, and pitch	0.48^***^	0.46^***^	0.40^***^	0.41^***^	0.13^***^	0.44^***^	0.58^***^
Speech, match pitch only	0.22^*^	0.22^*^	0.15	0.23^*^	0.18^*^	0.18^*^	0.35^***^
Music, match rhythm only	0.52^***^	0.56^***^	0.45^***^	0.50^***^	0.45^***^	0.45^***^	0.62^***^
Music, match rhythm, and pitch	0.54^***^	0.64^***^	0.57^***^	0.55^***^	0.51^***^	0.47^***^	0.67^***^
Music, match pitch only	0.43^***^	0.48^***^	0.39^***^	0.41^***^	0.41^***^	0.37^***^	0.55^***^
Tapping, 3/4 time	0.28^**^	0.22^*^	0.20^*^	0.20^*^	0.15	0.15	0.13
Vector length, 3/4 time	0.18^*^	0.19^*^	0.19^*^	0.25^**^	0.20^*^	0.21^*^	0.20^*^
Tapping, 4/4 time	0.23^*^	0.25^**^	0.18^*^	0.19^*^	0.21^*^	0.18^*^	−0.19^*^
Vector length, 4/4 time	0.14	0.16	0.10	0.08	0.14	0.02	0.20^*^

* *p < 0.05*,

** *p < 0.01*,

*** *p < 0.001*.

In order to explore the relative strength of associations between our music tasks, overall language development (receptive vs. expressive), phonological development and reading development, multiple regression analyses were used. To assess relationships for these four outcome measures, 4 sets of 3 equations were created, a set of equations for CELF Receptive language scores, a set for CELF Expressive language scores, a set for phonological awareness (rhyme oddity) and a set for reading (BAS single word reading standard scores) respectively. The predictors in the sets of equations were chosen from the task battery to be relatively independent measures of the variables of interest, and age and NVIQ were also included, to account for general developmental variance. The predictors in the first equation in each set were thus age, NVIQ, matching rhythm in speech and matching pitch in speech (AXB task scores, recall that performance in these two speech tasks was not related). The predictors in the second and third equations in each set were age, NVIQ, performance in the musical beat perception task, and performance in the tapping-to-music task (either measured via the SyncScore or by Vector Length in 4/4 time, the easier tempo). The bootstrap function in SPSS was applied in each case, as not all predictors were normally distributed in the full sample (1000 permutations, confidence intervals 95%, bias corrected and accelerated). The results are shown as Tables 7, 8, which report the Beta values with standard errors and confidence intervals, the standardized Beta values, and the bootstrapped *p*-values.

**Table 7:** **Multiple regression equations with receptive and expressive language scaled scores as dependent variables and age, NVIQ and the music/speech tasks as predictors**.

	**CELF recep ь (CI)**	**ь SE**	**ß**	**Sig**	**CELF expr ь (CI)**	**ь SE**	**ß**	**Sig**
NVIQ	0.169 (0.12 to 0.22)	0.03	0.55^**^	0.001	0.17 (0.12 to 0.22)	0.02	0.54^**^	0.001
Age	−0.02 (−0.09 to 0.05)	0.04	−0.06	0.59	−0.03 (−0.10 to 0.03)	0.03	−0.09	0.30
Speech rhythm	0.16 (0.02 to 0.29)	0.07	0.18^*^	0.029	0.14 (−0.01 to 0.30)	0.07	0.17^*^	0.048
Speech pitch	0.10 (0.00 to 0.20)	0.06	0.12	0.082	0.11 (−0.02 to 0.24)	0.06	0.14	0.099
NVIQ	0.17 (0.12 to 0.22)	0.03	0.55^**^	0.001	0.16 (0.11 to 0.21)	0.03	0.52^**^	0.001
Age	−0.01 (−0.07 to 0.06)	0.04	−0.02	0.86	−0.02 (−0.08 to 0.04)	0.03	−0.06	0.449
Musical beat	7.4 (0.47 to 13.6)	3.5	0.18^*^	0.039	7.80 (0.11 to 15.5)	3.7	0.18^*^	0.032
Tapping 4/4	0.37 (−0.35 to 1.1)	0.35	0.09	0.29	0.67 (−0.09 to 1.4)	0.38	0.16	0.085
VL 4/4	1.63 (−0.67 to 3.8)	1.14	0.11	0.14	2.18 (−0.019 to 4.5)	1.15	0.15	0.052

*CELF Recep, CELF Receptive Scaled Score; ь (CI), unstandardized beta and confidence intervals; ь SE—standardized error for ь; ß, standardized beta coefficient; Sig, bootstrapped significance; CELF Expre, CELF Expressive Scaled Score; Speech Rhythm, Match Speech Rhythm AXB score; Speech Pitch, Match Speech Pitch AXB score; Musical Beat, Musical Beat Perception accuracy; Tapping 4/4, timing accuracy when tapping to music in 4/4 time (synchronization score); VL 4/4, timing accuracy when tapping to music in 4/4 time, vector length score*.

* *p < 0.05*;

** *p < 0.01*.

**Table 8:** **Multiple regression equations with phonological awareness (Rhyme Oddity) and single word reading (BAS SS) as dependent variables and age, NVIQ and the music/speech tasks as predictors**.

	**Phon aware ь (CI)**	**ь SE**	**ß**	**Sig**	**BAS read ь (CI)**	**ь SE**	**ß**	**Sig**
NVIQ	0.10 (0.06 to 0.13)	0.02	0.44^**^	0.001	0.49 (0.33 to 0.64)	0.08	0.46^**^	0.001
Age	0.05 (0.00 to 0.10)	0.02	0.19^*^	0.033	−0.26 (−0.45 to −0.06)	0.10	−0.21^*^	0.013
Speech rhythm	0.16 (0.06 to 0.24)	0.05	0.26^**^	0.003	0.83 (0.33 to 1.3)	0.26	0.28^**^	0.001
Speech pitch	0.12 (0.05 to 0.18)	0.04	0.21^**^	0.002	0.46 (0.11 to 0.79)	0.18	0.17^*^	0.015
NVIQ	0.09 (0.06 to 0.13)	0.02	0.41^**^	0.001	0.50 (0.36 to 0.67)	0.08	0.48^**^	0.001
Age	0.06 (0.02 to 0.10)	0.02	0.24^**^	0.005	−0.21 (−0.40 to 0.03)	0.11	−0.17	0.051
Musical beat	9.6 (3.7 to 14.8)	2.8	0.32^**^	0.001	36.4 (1.1 to 57.5)	13.2	0.25^*^	0.005
Tapping 4/4	0.29 (−0.23 to 0.82)	0.26	0.10	0.266	1.4 (−1.2 to 3.9)	1.2	0.10	0.239
VL4/4	0.612 (−0.887 to 2.56)	0.90	0.058	0.505	1.6 (−5.9 to 1.5)	4.3	0.031	0.708

*Phon Aware, oddity rhyme task; ь (CI), unstandardized beta and confidence intervals; ь SE—standardized error for ь; ß, standardized beta coefficient; Sig, bootstrapped significance; BAS Read, BAS Single Word Reading Scaled Score; Speech Rhythm, Match Speech Rhythm AXB score; Speech Pitch, Match Speech Pitch AXB score; Musical Beat, Musical Beat Perception accuracy; Tapping 4/4, timing accuracy when tapping to music in 4/4 time (synchronization score); VL 4/4, timing accuracy when tapping to music in 4/4 time, vector length score*.

* *p < 0.05*;

** *p < 0.01*.

The regression analyses for children's receptive language scores (see Table 7) accounted for a significant 68% [*F*_(4, 90)_ = 18.8, *p* < 0.001], 67% [*F*_(4, 89)_ = 17.6, *p* < 0.001] and 68% [*F*_(4, 88)_ = 19.0, *p* < 0.001] of the variance respectively. The first equation revealed two significant predictors, NVIQ and matching rhythm in speech. SLI children with higher NVIQ and better rhythm matching had better receptive language outcomes. The second and third equations also revealed two significant predictors, NVIQ and musical beat perception accuracy. SLI children with higher NVIQ and better performance in the musical beat perception task also had better receptive language outcomes. For expressive language scores (also Table 7), the analyses accounted for a significant 67% [*F*_(4, 90)_ = 17.9, *p* < 0.001], 68% [*F*_(4, 89)_ = 18.8, *p* < 0.001], and 68% [*F*_(4, 88)_ = 18.7, *p* < 0.001] of the variance respectively. The first equation again revealed two significant predictors, NVIQ and matching rhythm in speech, and the second and third equations again revealed two significant predictors, NVIQ and performance in the musical beat perception task. In each case, SLI children with higher NVIQ and better rhythmic performance had higher expressive language outcomes. Inspection of Table 7 shows that performance in the tapping-to-music task approached significance as a predictor of scores in the expressive language measure (*p's* = 0.085 and 0.052). Better motor synchronization to the beat related to better language outcomes.

Regarding phonological awareness (Table 8), each equation accounted for a significant 67% of the variance [*F*_(4, 90)_ = 18.6, *p* < 0.001, *F*_(4, 89)_ = 18.3, *p* < 0.001], [*F*_(4, 88)_ = 17.6, *p* < 0.001]. The first equation revealed that all four independent variables were significant predictors of phonological awareness, with older children with higher NVIQ and better rhythm and pitch matching showing better phonological awareness. In the second and third equations, the significant predictors of phonological awareness were age, NVIQ and musical beat perception. Older children with higher NVIQ and better performance in the musical beat perception task showed better phonological awareness. The tapping-to-music task did not approach significance as a predictor of phonological development in this sample for either measure (SyncScore or Vector Length). Finally, for single word reading (BAS SS, also Table 8), the equations accounted for a significant 70% of the variance [*F*_(4, 90)_ = 21.6, *p* < 0.001], 68% of the variance [*F*_(4, 89)_ = 19.2, *p* < 0.001] and 67% of the variance [*F*_(4, 88)_ = 18.1, *p* < 0.001] respectively. All four independent variables were again significant predictors in the first equation, while the second and third equations showed two significant predictors, NVIQ and musical beat perception, with age close to significant (*p* = 0.051). Children with higher NVIQ, better rhythm and pitch matching performance and better musical beat perception showed better reading development. However, age was negatively related to reading development. This most likely reflects the fact that reading scores tend to plateau in older SLI children. Performance in the tapping task (4/4 time) was not significantly related to reading development. Hence the only independent variables that were *consistent predictors* of performance across the language, phonology and reading outcome measures were the measures of sensitivity to rhythm in speech and the musical beat perception task. Accordingly, perceptual awareness of rhythm patterns in speech and in music appears to be an important predictor of individual differences in language, phonology and reading development. However, some of the experimental tasks used here are more sensitive measures of this relationship than others.

## Discussion

Here we set out to investigate sensitivity to rhythm in music and speech for children with SLIs. Following prior studies of children with dyslexia (e.g., Huss et al., 2011; Goswami et al., 2013; Flaugnacco et al., 2014) and language impairments (e.g., Przybylski et al., 2013; Cumming et al., 2015), we expected to find rhythm perception deficits in children with SLIs. We were also interested in whether sensitivity to rhythm in music might be stronger than sensitivity to rhythm in speech for children with speech and language difficulties, in which case musical interventions might be of benefit (musical interventions may enhance shared cognitive/neural resources; see Goswami, 2012a,b; Przybylski et al., 2013; Gordon et al., 2015). The data revealed significant impairments in processing rhythm in the children with SLIs, but some tasks were more sensitive to the children's difficulties than other tasks. Contrary to expectation, the music AXB tasks were only easier than the matched speech AXB tasks for typically-developing children. The children with SLIs found it difficult to make rhythm judgements in *both* speech and music.

At the group level, the children with SLIs who had intact NVIQ did not show significant impairments in a musical beat perception task previously administered to children with developmental dyslexia (Huss et al., 2011; Goswami et al., 2013), although both the Pure SLI and SLI PPR groups scored more poorly than their TD controls. When IQ varied, the children with SLIs (61%) were significantly poorer than TD controls (71%). Nevertheless, the *relative* performance of the children with SLI studied here compared to TD controls showed less impairment compared to children with dyslexia. Comparison with prior dyslexic performance (Huss et al., 2011) reveals that older children (10-year-olds) with dyslexia and intact NVIQ averaged 63% correct in this task, while their TD controls averaged 84% correct (Huss et al., 2011). This may be suggestive of a less severe impairment in children with SLI in perceiving patterns of musical beats in this task, although longitudinal data are required. Nevertheless, individual differences in the musical beat perception task were a significant predictor of both the expressive and receptive language scores achieved by SLI children in multiple regression equations (see Table 7). Individual differences in musical beat perception were also a significant predictor of individual differences in both phonological awareness and reading development (Table 8). The latter developmental relationship is also found for children with dyslexia (Huss et al., 2011; Goswami et al., 2013).

In a tapping-to-music task created for the current study (in 3/4 time and 4/4 time), children with Pure SLI and intact IQ did show a significant impairment compared to TD children, for both tempi and for both synchronization measures (SyncScore, vector length). The group effect also approached significance for children with SLIs and phonological impairments (the SLI PPR group, *p* = 0.09 for SyncScores and *p* = 0.12 for vector length). When the whole sample was considered (IQ varying), the group effect was significant for the SyncScore measure (*p* = 0.002) but not for the vector length measure (3/4 time, *p* = 0.096; 4/4 time, *p* = 0.113). Overall, the data suggest that impaired motor synchronization to the beat is characteristic of children with SLIs and does not reflect low NVIQ. This finding supports our prior data showing motor variability in synchronization to the beat in children with SLIs as well as the data of others (e.g., Corriveau and Goswami, 2009; Woodruff Carr et al., 2014). However, most previous demonstrations of significant rhythmic tapping deficits in children with SLIs have utilized a metronome or drumbeat, where the pulse rate is clear. Our music task required synchronization to the pulse rate underlying the different musical instruments, potentially providing richer support to scaffold children's rhythmic timing accuracy (in music there are more auditory cues supporting the beat). The finding that motor synchronization to the beat in SLI children can be impaired for rich musical stimuli as well as for a metronome beat (cf. Corriveau and Goswami, 2009) suggests that musical interventions *per se* may not have much utility in enhancing neural entrainment to language (syllable) beats for children with SLIs. Rather, interventions may have to consider carefully how the musical beat supports the *prosodic phrasing* of target linguistic utterances.

Unexpectedly, individual differences in the tapping-to-music tasks did not reach significance as predictors of receptive language development in this cohort of children (Table 7), although individual differences did approach significance as predictors of expressive language development. This contrasts with some previous findings regarding tapping to a metronome (e.g., Corriveau and Goswami, 2009; Tierney and Kraus, 2013). Rhythmic timing accuracy as measured by this music task also failed to reach significance as a predictor of phonological awareness and reading (Table 8). These findings differ from the associations between beat-based timing accuracy and phonological and reading development that we have found in samples of children and adults with developmental dyslexia Thomson et al., 2006; Thomson and Goswami, 2008; see also Tierney and Kraus, 2013). One possibility is that the inconsistency in findings arises from our use of musical pieces rather than a metronome to measure variability in motor synchronization. Corriveau and Goswami (2009) did find significant relationships between tapping variability, phonology and reading in a different SLI cohort. Alternatively, the difference may arise because children with SLIs are relatively less impaired than children with dyslexia in motor synchronization tasks.

We also created a novel music AXB task for this study which required participants to match tunes to a filtered standard tune (X) on the basis of shared rhythm patterns and/or shared pitch contour. A speech analog of the musical AXB task enabled direct comparison with children's sensitivity to rhythm patterns and pitch contours in speech. The analyses showed that all children, TD and SLI, found matching on the basis of rhythm easier than matching on the basis of pitch, for both music and speech. The music tasks were also easier than the speech tasks for the TD children, but not for the children with SLIs. In fact, the children with SLIs showed poorer performance in all the rhythm tasks than the TD children, although performance was only significantly poorer for the SLI PPR group and the whole SLI group with NVIQ varying (the group effect for the Pure SLI children did not reach significance, *p* = 0.16). As would be expected, individual differences in the speech AXB rhythm task were a significant predictor of receptive and expressive language development (Table 7). In contrast, individual differences in the speech AXB pitch contour task did not predict language outcomes (Table 7). Individual differences in the speech AXB pitch contour task did, however, predict phonological and reading outcomes (Table 8).

Finally, we turn to the possible benefits of musical interventions for remediating the language deficits in children with SLIs. The fact that the children with SLIs found the music AXB tasks more difficult than the speech AXB tasks was unexpected. It suggests that musical interventions for children with SLIs need to be very carefully designed if they are to be effective. Given that the linguistic difficulties in SLI are associated with prosodic structure, it seems important that future studies investigate whether most benefit might be derived from musical sequences that match the *overall prosodic phrasing* of speech utterances, so that music is used to highlight precedence and prominence relations in larger lexical structures (Frazier et al., 2006). Simple beat synchronization tasks *per se* may not offer the same benefits for children with SLIs that are found for children with developmental dyslexia. For children with reading difficulties, phonological benefits ensue from music-based remediation that focuses on multi-modal synchronization to the beat (e.g., synchronizing spoken stressed syllables with clapping or marching actions and with a musical accompaniment, see Bhide et al., 2013). In Bhide et al.'s study, the degree of increased efficiency of motor synchronization to the beat by the children (temporal accuracy in bongo drumming to different rhythms) was significantly related to individual gains in reading. Temporal sampling theory provides a potential neural cross-modal explanation of this finding based on phase-phase coupling at delta and theta rates (Goswami, 2011, 2015). This relationship has yet to be tested for children with SLIs regarding individual gains in language. In cases of Pure SLI, where the child has morphological but not phonological deficits, motor synchronization to the beat *per se* may not offer significant benefits for grammatical development. More data is required to find out whether remediation for children with SLIs should focus primarily on the auditory and motor domains, as in developmental dyslexia (Bhide et al., 2013; Tierney and Kraus, 2013).

Indeed, *multi-modal* rhythmic interventions for children with SLIs (including the visual modality) may potentially offer greater benefits than simpler beat-based interventions. Children with SLIs typically have deficits in the auditory processing of both ART and duration. Grouping cues, such as those used to group precedence and prominence relations in an utterance, may rely more on sensitivity to duration. This has yet to be systematically investigated, but it is interesting to consider that SLI children's attention to “visual prosody” (the mouth, jaw, cheek, and head movements that the speaker unconsciously produces when emphasizing oral prosody, see Munhall et al., 2004) has not been quantified. Visual prosody may be important developmentally in supporting the long-range aural perception required to identify the weaker syllables that typically carry morphological information (Ghazanfar and Takahashi, 2014a,b). Attention to visual prosody is thus potentially crucial for morphological development in children. If this were to be the case, then musical interventions that involve group singing, or other musical activities that offer opportunities for visual as well as auditory and motor rhythmic synchronization, may offer the best outcomes in remediating language, syntax and phonology in children with SLIs.

### Conflict of interest statement

The authors declare that the research was conducted in the absence of any commercial or financial relationships that could be construed as a potential conflict of interest.
