# Supplementary material for: Awareness of Rhythm Patterns in Speech and Music in Children with Specific Language Impairments
Source: Front Hum Neurosci. 2015 Dec 22;9:672. doi: 10.3389/fnhum.2015.00672 (PMC4686839; doi:10.3389/fnhum.2015.00672)
Supplement: Supplementary file 1 [file DataSheet1.docx]

1. **Appendix. A 1 – Musical ‘Rhythm only’ stimuli**

| **Trial number** | **Girl in green (A)** | **Girl in blue (B)** |
| --- | --- | --- |
| Practice1 | SSW**WS** | SSW**SW** |
| Practice2 | **W**WSWSW | **S**WSWSW |
| 1 | SSW**SW** | SSW**WS** |
| 2 | WSW**W**WSW | WSW**S**WSW |
| 3 | SSWS**S** | SSWS**W** |
| 4 | WWS**W**WS | WWS**S**WS |
| 5 | WWS**S**WS | WWS**W**WS |
| 6 | WSW**S**WSW | WSW**W**WSW |
| 7 | SSWS**W** | SSWS**S** |
| 8 | WWSW**WS** | WWSW**SW** |
| 9 | **S**WSWSW | **W**WSWSW |
| 10 | SSW**W**S | SSW**S**S |
| 11 | **W**WSWWSW | **S**WSWWSW |
| 12 | SSW**S**S | SSW**W**S |
| 13 | **S**WSWWSW | **W**WSWWSW |
| 14 | WWSW**SW** | WWSW**WS** |
| - ***Bold indicates difference between A and B*** - ***Underlined indicates which stimulus matched stimulus X*** | | |

**A2 – Musical ‘Pitch only’ stimuli**

| **Trial number** | **Girl in green (A)** | **Girl in blue (B)** |
| --- | --- | --- |
| Practice1 | G F^#^ G **E B** | G F^#^ G **B E** |
| Practice2 | **F^#^ E** A A G E | **E F^#^** A A G E |
| 1 | E F^#^ G A **G E** | E F^#^ G A **E G** |
| 2 | D G D **D E** | D G D **E D** |
| 3 | E G A **G B** E D | E G A **B G** E D |
| 4 | B^b^ F F **G A** B^b^ | B^b^ F F **A G** B^b^ |
| 5 | F^#^ G F^#^ **B E** | F^#^ G F^#^ **E B** |
| 6 | E B A **A B** | E B A **B A** |
| 7 | **E F** F G A G F | **F E** F G A G F |
| 8 | G D F **E D** | G D F **D E** |
| 9 | **G A** F E F C | **A G** F E F C |
| 10 | F^#^ A B **A F^#^** D | F^#^ A B **F^#^ A** D |
| 11 | D G G **G F^#^** F^#^ D | D G G **F^#^ G** F^#^ D |
| 12 | F F A F **A B^b^** | F F A F **B^b^ A** |
| 13 | **B E** F^#^ G F E D | **E B** F^#^ G F E D |
| 14 | G G E **B A** | G G E **A B** |
| - ***Bold indicates difference between A and B*** - ***Underlined indicates which stimulus matched stimulus X*** | | |

**A3 – Musical ‘Rhythm and pitch’ stimuli**

| **Trial number** | **Girl in green (A)** | **Girl in blue (B)** | **Melody** |
| --- | --- | --- | --- |
| Practice1 | SSW**SW** | SSW**WS** | F^#^ G F^#^ B E |
| Practice2 | **S**WSWSW | **W**WSWSW | A G F E F C |
| 1 | **S**WSWWSW | **W**WSWWSW | B E F^#^ G F E D |
| 2 | SSW**W**S | SSW**S**S | G G E B A |
| 3 | WWS**S**WS | WWS**W**WS | F^#^ A B A F^#^ D |
| 4 | **W**WSWWSW | **S**WSWWSW | F E F G A G F |
| 5 | WWSW**SW** | WWSW**WS** | E F^#^ G A G E |
| 6 | **W**WSWSW | **S**WSWSW | F^#^ E A A G E |
| 7 | SSW**S**S | SSW**W**S | D G D E D |
| 8 | WWS**W**WS | WWS**S**WS | B^b^ F F G A B^b^ |
| 9 | WSW**S**WSW | WSW**W**WSW | D G G F^#^ G F^#^ D |
| 10 | SSW**WS** | SSW**SW** | E B A B A |
| 11 | SSWS**S** | SSWS**W** | G D F E D |
| 12 | WWSW**WS** | WWSW**SW** | F F A F A B^b^ |
| 13 | SSWS**W** | SSWS**S** | G F^#^ G B E |
| 14 | WSW**W**WSW | WSW**S**WSW | E G A B G E D |
| - ***Bold indicates difference between A and B*** - ***Underlined indicates which stimulus matched stimulus X*** | | | |

**A4 – Speech ‘Rhythm only’ stimuli**

| **Trial number** | **Lady in green (A)** | **Lady in purple (B)** |
| --- | --- | --- |
| Practice1 | Can you see the **white**board? | Do you like **pine**apple? |
| Practice2 | I can play the **flute** | It’s **time** they went home |
| 1 | My **friend** is coming to stay | Let’s play on the com**pu**ter |
| 2 | Shall we watch a **film**? | Are you a**wake** now? |
| 3 | Shall we go on a **bike** ride? | Can the teacher hear the **class**? |
| 4 | Let’s play on the com**pu**ter | My **friend** is coming to stay |
| 5 | I **like** the colour red | It’s good to take a **walk** |
| 6 | Is it **far** to town? | Shall we watch a **film**? |
| 7 | Can the teacher hear the **class**? | Shall we go on a **bike** ride? |
| 8 | Does the bus come on **time**? | Can you see the **white**board? |
| 9 | You draw nice **pic**tures | I can play the **flute** |
| 10 | We don’t have to **wo**rry | I **like** the colour red |
| 11 | Are you a**wake** now? | Is it **far** to town? |
| 12 | Do you like **pine**apple? | Does the bus come on **time**? |
| 13 | It’s **time** they went home | You draw nice **pic**tures |
| 14 | It’s good to take a **walk** | We don’t have to **wo**rry |
| - ***Bold indicates difference between A and B*** - ***Underlined indicates which stimulus matched stimulus X*** | | |

**A5 – Speech ‘Pitch only’ stimuli**

| **Trial number** | **Lady in green (A)** | **Lady in purple (B)** |
| --- | --- | --- |
| Practice1 | Are we an**noy**ing you? | Were **ma**ny men around? |
| Practice2 | **Your** meal will arrive warm | We were relying on **mum** |
| 1 | Were many **men** around? | Am **I** yelling my name? |
| 2 | **You** rarely worry | I learn on my **own** |
| 3 | Why are we wearing **ye**llow? | **When** will we know any more? |
| 4 | Why are you **ear**ly? | Are **you** kneeling now? |
| 5 | Am I yelling my **name**? | Will **you** ring me one year? |
| 6 | An **owl** lay on a wall | **I** love running around |
| 7 | We **may** win a million | Your meal **will** arrive warm |
| 8 | Will you ring **me** one year? | Are we annoying **you**? |
| 9 | When will we know any **more**? | **Why** are we wearing yellow? |
| 10 | **I** learn on my own | You rarely **wo**rry |
| 11 | I **love** running around | An owl **lay** on a wall |
| 12 | **My** yoyo will roll away | We may win a **mi**llion |
| 13 | Are you kneeling **now**? | Why are **you** early? |
| 14 | **We** were relying on mum | My yoyo will roll a**way** |
| - ***Bold indicates difference between A and B*** - ***Underlined indicates which stimulus matched stimulus X*** | | |

**A6 – Speech ‘Rhythm and pitch’ stimuli**

| **Trial number** | **Lady in green (A)** | **Lady in purple (B)** |
| --- | --- | --- |
| Practice1 | Can we go **swim**ming? | Does it **rain** a lot? |
| Practice2 | **Les**sons are sometimes fun | We’re going to the **shops** |
| 1 | It’s hard to see the **TV** | You need to do your **home**work |
| 2 | Does it **rain** a lot? | Is it time for **lunch**? |
| 3 | Shall we go on **Mon**day? | Are you ready for **school**? |
| 4 | You need to do your **home**work | It’s hard to see the **TV** |
| 5 | Is it time for **lunch**? | Can we go **swim**ming? |
| 6 | We like playing **games** | I **like** baking cakes |
| 7 | We’re going to the **shops** | You can take a **break** soon |
| 8 | Are you ready for **school**? | Is it **sun**ny outside? |
| 9 | Let’s kick the **foot**ball | We like playing **games** |
| 10 | Are the holidays **bor**ing? | Do you want a glass of **juice**? |
| 11 | Is it **sun**ny outside? | Shall we go on **Mon**day? |
| 12 | You can take a **break** soon | **Les**sons are sometimes fun |
| 13 | I **like** baking cakes | Let’s kick the **foot**ball |
| 14 | Do you want a glass of **juice**? | Are the holidays **bor**ing? |
| - ***Bold indicates difference between A and B*** - ***Underlined indicates which stimulus matched stimulus X*** | | |
